# Actively forming microbial mats provide insight into the development of microdigitate stromatolites

**DOI:** 10.1038/s41598-025-90175-0

**Published:** 2025-02-14

**Authors:** Judit Makk, Ábel Csongor Németh, Erika Tóth, Péter Németh, Ivett Kovács, Attila Demény, György Sipos, Andrea K. Borsodi, Nóra Tünde Lange-Enyedi

**Affiliations:** 1 Department of Microbiology, ELTE Eötvös Loránd University, Pázmány P. Sétány 1/C, 1117 Budapest, Hungary; 2 Department of Public Health Laboratories, National Public Health and Pharmaceutical Center, Albert Flórián Street 2-6, 1097 Budapest, Hungary; 3 Doctoral School of Environmental Sciences, ELTE Eötvös Loránd University, Pázmány Péter Street 2, 1117 Budapest, Hungary; 4 Institute for Geological and Geochemical Research, HUN-REN Research Centre for Astronomy and Earth Sciences, Budaörsi Út 45, 1112 Budapest, Hungary; 5 Research Institute of Biomolecular and Chemical Engineering, Nanolab, University of Pannonia, Egyetem Út 10, 8200 Veszprém, Hungary; 6 Functional Genomics and Bioinformatics Group, Faculty of Forestry, University of Sopron, Bajcsy-Zsilinszky Út 4, 9400 Sopron, Hungary; 7 Institute of Aquatic Ecology, HUN-REN Centre for Ecological Research, Karolina Út 29, 1113 Budapest, Hungary

**Keywords:** Amplicon sequencing, Bacteria biodiversity, Biofilm, Thermal well, Microdigitate stromatolites, Ecology, Microbiology, Biogeochemistry

## Abstract

Stromatolites can be traced back to ∼3.5 billion years. They were widespread in the shorelines of ancient oceans and seas. However, they are uncommon nowadays, and basic information is lacking about how these unique carbonate structures developed. Here we study the unusually thick (3–5 cm) biofilms of the 79.2 °C outflow from Köröm thermal well (Hungary) and demonstrate that its microbial mat – carbonate architecture is similar to fossilized microdigitate stromatolites. Our observations reveal vertically oriented fibrous mineral fabrics, typical of stromatolites, in the red biofilm and clotted mesostructures, typical of thrombolites, in the green biofilm. These layers contain carbonate peloids and show network structures, formed by filamentous microbes. The 16S rRNA gene-based amplicon sequencing implies that numerous undescribed taxa may contribute to the carbonate mineralisation. The biofilms abundantly contain the phyla Bacteroidota, Pseudomonadota and Cyanobacteria. *Geitlerinema* PCC-8501 and *Raineya* are characteristic for the green biofilm, whereas uncultured Oxyphotobacteria, unc. Saprospiraceae and unc. Cytophagales are abundant in the red biofilm. A hydrogen-oxidizing *Hydrogenobacter* within the phylum Aquificota and unclassified Bacteria together with the phylum Deinococcota dominate the water and carbonate samples. The morphological structure and taxonomic composition of Köröm biofilm is a unique representation of the development processes of microbialite formations.

## Introduction

Microbial mats are unique type of biofilms that typically develop in the liquid–solid interface of a broad range of environments including extreme aquatic environments such as hot springs, deep-sea vents, cold seeps, alkaline and hypersaline lakes^1–3^. The thicknesses of microbial mats range from several millimetres to a few centimetres^4^. They consist of several multicolour layers, and their formation is highly dependent on the environmental parameters such as sunlight, humidity, pH, salinity, temperature and available nutrients (electron donors and acceptors). The characteristic multilayered structure of microbial mats results from the physicochemical gradients created and maintained by the activities of members of microbial communities^5^. Mats formed in sunny surface environments usually have an upper oxic layer, dominated by oxygen-producing phototrophs (e.g. Cyanobacteria)^6,7^ that are simultaneously responsible for the primary production, nitrogen fixation and shading of the deeper layer from solar radiation. The deeper anoxic layers are usually inhabited by anaerobic chemoorganotrophs, chemolithotrophs such as sulphate-reducing bacteria and archaea. By producing large amounts of extracellular polymeric substances (EPS), microorganisms can establish various interactions by embedding themselves in the biofilm^4^.

The complex interactions between microbial mats and their geochemical environment can potentially result in the precipitation of authigenic minerals including carbonates such as calcite, aragonite and dolomite, as well as silicates, sulphates and iron-oxides^6,8^. Biofilms can also absorb elements including metalloids, arsenic, etc., suggesting the involvement of specific adaptation mechanisms, such as arsenate respiration^9^. These accreted and lithified microbial mats form organosedimentary deposits referred to as microbialites^10,11^. Based on the millimetre- to centimetre-sized (mesoscopic) minerals occurring inside the microbial mats, the microbialites can be distinguished into four groups: i) stromatolites consisting of laminated mesostructure of minerals, ii) dendrolites consisting of dendritic, lamina-free mesostructure of minerals, iii) thrombolites consisting of clot like, lamina-free mineral fabric and iv) leiolites consiting of an aphanitic carbonate fabric^11^. Stromatolites and thrombolites are the most common living forms^6^.

Carbonate precipitation in thermal springs is mainly induced by CO_2_ degassing of supersaturated calcium bicarbonate-rich waters. However, in warm (20–40 °C) and mesothermal (40–75 °C) springs cyanobacteria and many other bacteria can also mediate the carbonate precipitation via a combination of physico-chemical, biologically induced and influenced processes associated with the microbial mats^12^. Microbial metabolic pathways, such as photosynthesis, ureolysis, ammonification, denitrification, sulphate reduction and methane oxidation, seem to play a key role in carbonate precipitation by modifying the microenvironment and increasing alkalinity and dissolved inorganic carbon (DIC)^13^. The properties of the EPS matrix influence the organomineralisation through cation binding/release and by providing mineral nucleation sites, either inhibiting or promoting carbonate precipitation^6^. The possibility that microorganisms contribute to mineral precipitation at hot springs was first explored in Yellowstone National Park^14,15^. Microbially mediated carbonate formation is an important part of the global carbon cycle and has the potential to sequester large amounts of CO_2_. Most photosynthetic microbialite communities are carbonate-rich, so they constitute carbon reservoirs in the form of both biomass and carbonates^16^. Due to their complex nature, the exact role of microbial communities in mineral precipitation and dissolution processes is not fully clarified.

Nowadays, actively forming microbialites can mainly be found in restricted environments, characterized by high chloride or high sulphide contents, strong irradiation or extremely high temperatures (from 60 °C upwards). Examples can be found in a great variety of environments including geothermal springs^17–20^, alkaline^21,22^, hypersaline^3,23–25^ and marine^26–28^ aquatic habitats and caves^29^. Microbialites are very vulnerable ecosystems. They are becoming increasingly exposed to the effects of global climate change, i.e., warmer temperatures, rising sea levels and ocean acidification^27,30^. Microbialites can be dissolved by acidic water or eroded by wind and waves. The study of living microbialites offers a unique opportunity to explore how these ecosystems interact with and adapt to environmental changes^31^.The understanding of modern microbialites as early microbial life terrestrial analogues has been of great scientific interest over the past decade, both from an ecological and evolutionary points of view^32^.

### Study location and thermal well characteristics

**Fig. 1:**
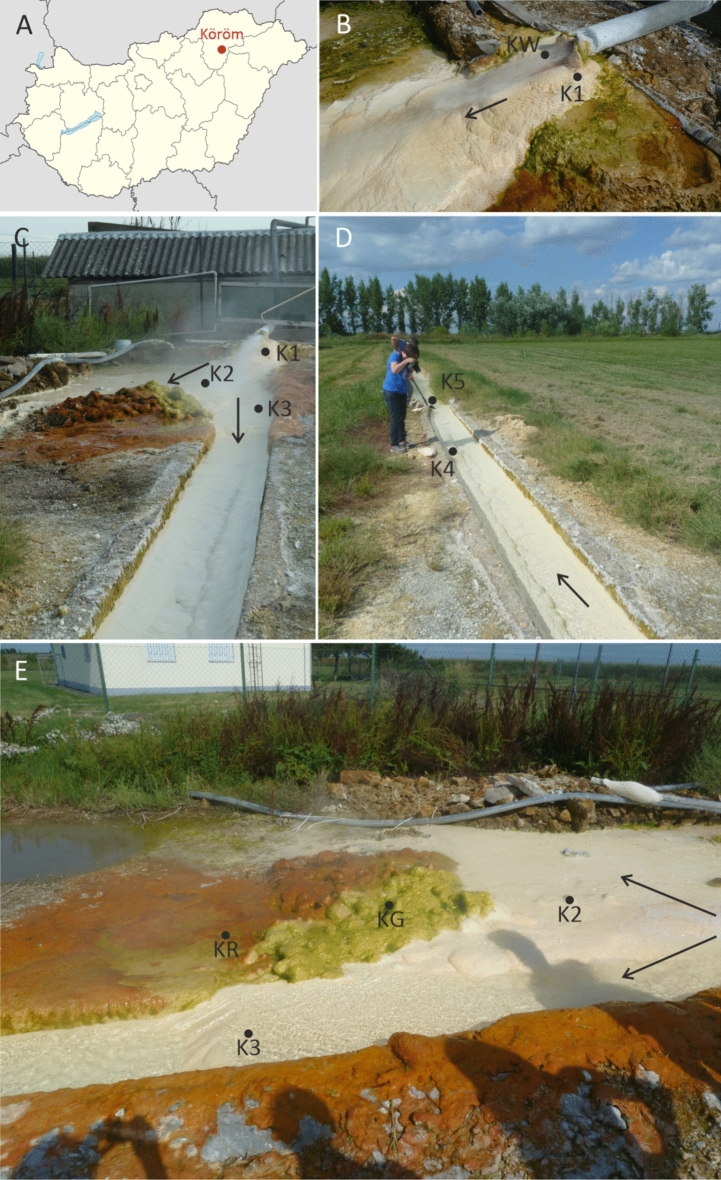
Map of the sampling site (**A**) and photos of the sampling points (**B**-**E**): the thermal water flowing from the wellhead through a pipe and sampling points KW, K1, K2 and K3 (**B** and **C**); the thermal water outflow channel and sampling points K4 and K5 (**D**); sampling points KR, KG, K2 and K3 (**E**). The K2 sampling site was ~ 0.5 m about half a metre from the edge of the dome, which was covered with green and red biofilms. The black arrows mark main flow paths.

The Köröm thermal well (Fig. 1, GPS coordinates N 47.997046° E 20.987198°) is one of the Hungarian hot springs, where intensive aragonite travertine precipitation occurs^33^. We have chosen this thermal well, because multilayered red and green mucilaginous calcium carbonate containing biofilms develope on the slopes of the travertine dome in significant quantities. It represents a natural laboratory where carbonate precipitation can be observed in a still-developing structured biofilm on the air–water interface of ~ 80 °C thermal water. The Köröm karst water well was drilled in 1953 during hydrocarbon exploration. Lénárt and Darabos^34^ suggested that the water of thermal water wells, located south-east of the Bükk Montains (in the vicinity of the Köröm well), derives from the Bükk Montains karstic water system that deeply infiltrated the underlying sediments. This infiltrating rainwater from the Bükk Mountains descends to a depth of nearly 2000 m, and it travels towards the plain areas, where it heats up. The water temperature at the bottom of the 1880 m deep well is 108 °C^33^. The thermal karst water contains elevated amounts of arsenic (403–424 µg/L) in the form of arsenous acid and dihydrogen arsenite, as well as boron (> 4.5 mg/L) and iron (> 100 µg/L). The arsenic content of the water has been proposed to have Earth´s crust origin^35^. Erőss et al.^36^ demonstrated that the water has an elevated radium content (105 mBq/L).

Here we study these multilayered structures and explore the relationships among the thermophilic microbial diversity, physical–chemical factors and carbonate precipitation using light microscopy, scanning and transmission electron microscopy (SEM, TEM) and X-ray diffraction (XRD) analysis. To assess the prokaryotic taxonomic diversity of well water, carbonate precipitates and biofilm samples, next-generation amplicon sequencing of the V3-V4 region of the 16S rRNA gene was applied. In addition, the morphology of the carbonate biofilms was compared with fossil and active microbialites of similar thermal water environments.

## Results

### Physical and chemical characterization of the thermal water

Starting from the wellhead, five sampling points (K1-K5) were selected along the drainage channel (Fig. 1). The water temperature in the drainage channel dropped steeply away from the well, but remained consistently above 60 °C. The conductivity also decreased from 1554 to 1515 µS/cm (Supplementary Table S1 online). The high temperature and the high amounts of dissolved inorganic compounds at the sampling points made in situ pH measurement challenging. Both Kele et al.^33^ and Mihucz et al.^35^ reported a pH value of 6.8 at the wellhead. An analogue travertine deposit occurs at Egerszalók, located about 50 km to the west from Köröm, at the eastern flanks of the Bükk Mts. The Egerszalók travertine is also formed from a thermal water well with a spring temperature of 66 °C and has a pH value of about 6.4. Its temperature decreases to 60 °C and its pH increases to ~ 7.2 at a distance of ~ 20 m from the wellhead^37^, which matches the distance between sampling points of K1 and K5 of this study. Based on this similarity, we presume the pH of sample point K5 also increased > 7. The transportation and sample storage made the laboratory chemical analyses uncertain due to irreversible precipitation of dissolved compounds, hence our water chemical data is only informative. Secondary precipitation was indicated by the low Ca^2+^ contents determined in samples K1 and K5 (18 and 7 mg/L, respectively), but such precipitation did not affect Na^+^ content. In fact, there is a good agreement in the measured Na^+^ contents of this study (330 to 339 mg/L) and previous analyses of the same drainage Sect. (337 to 340 mg/L, József Deák, unpublished results). The significant calcium carbonate deposition indicate a Ca^2+^-HCO_3_^–^–dominated solution. Its elevated Na^+^ concentration (> 330 mg/L) compared to the 59.5 mg/L Na^+^ concentration at Egerszalók ^37^ suggests that Köröm thermal well contains high amount Na^+^, presumbaly in the form NaHCO_3_.

### Morphology and mineral composition of the biofilm samples

The spring water flows and splashes almost continuously, supporting the growth of extensive microbial mats that form just below the air–water interface (splash zones) on top of the carbonate precipitates along the drainage channel (Fig. 1). At a distance of 5–6 m from the wellhead, brightly red and green coloured biofilm covers the surface of slightly prominent, previously precipitated carbonate domes in the immediate vicinity of the main flow. The green biofilm (KG) is visible on the surface of the carbonate dome exposed to splashing water, while the red biofilm (KR) occurs in a more sheltered area behind the domes (Fig. 1C and E).

**Fig. 2:**
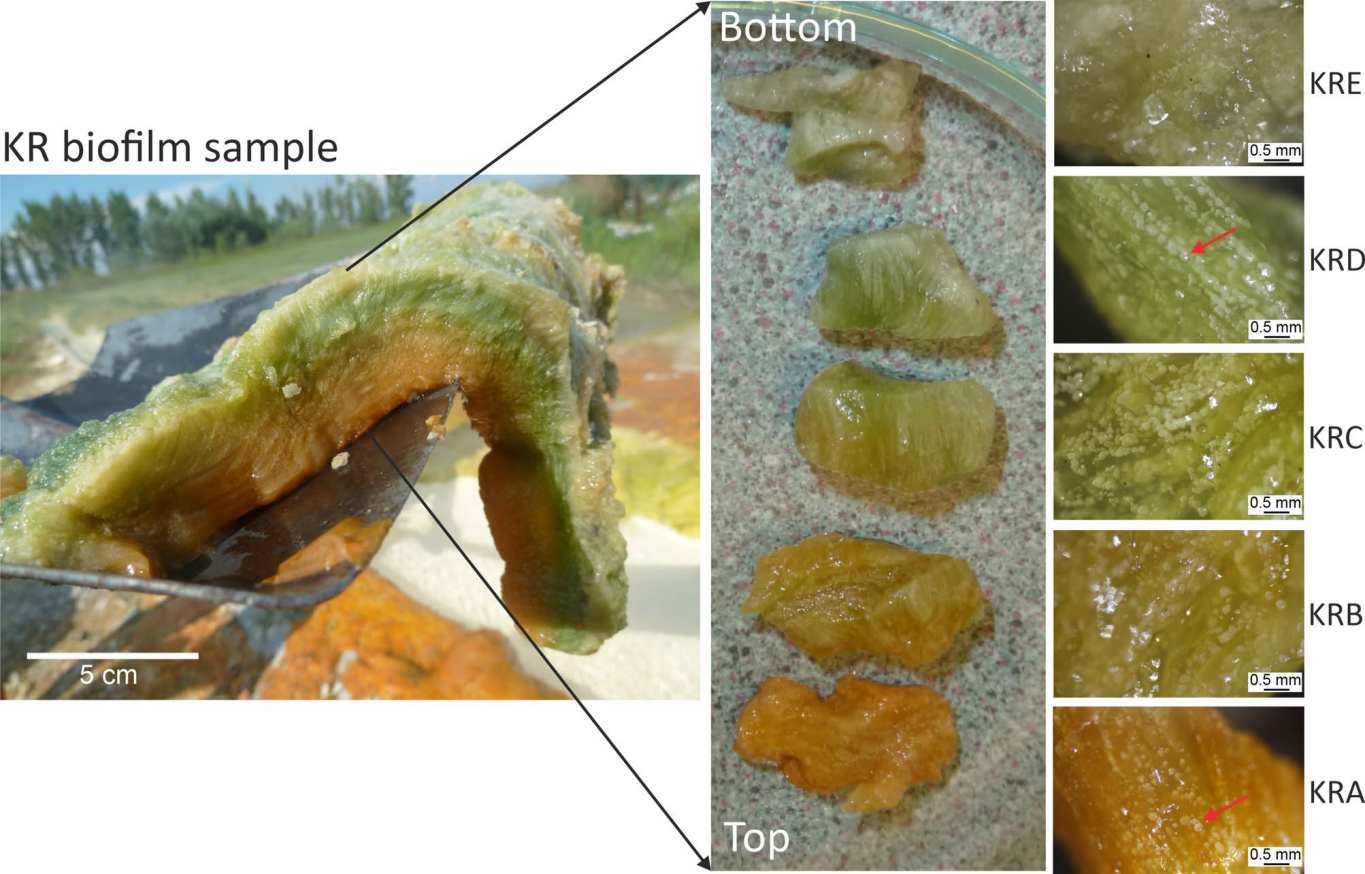
The Köröm thermal well red [KR] biofilm sample and its five isolated layers. Close-up photo of the red biofilm sample collected upside down with a shovel (left), the photos (middle) and stereomicroscope images (right) show disassembled biofilm layers. From top to bottom: red [KRA]; pale red [KRB]; pale green [KRC]; green [KRD]; greyish green [KRE]. The red arrows mark carbonate precipitates.

**Fig. 3:**
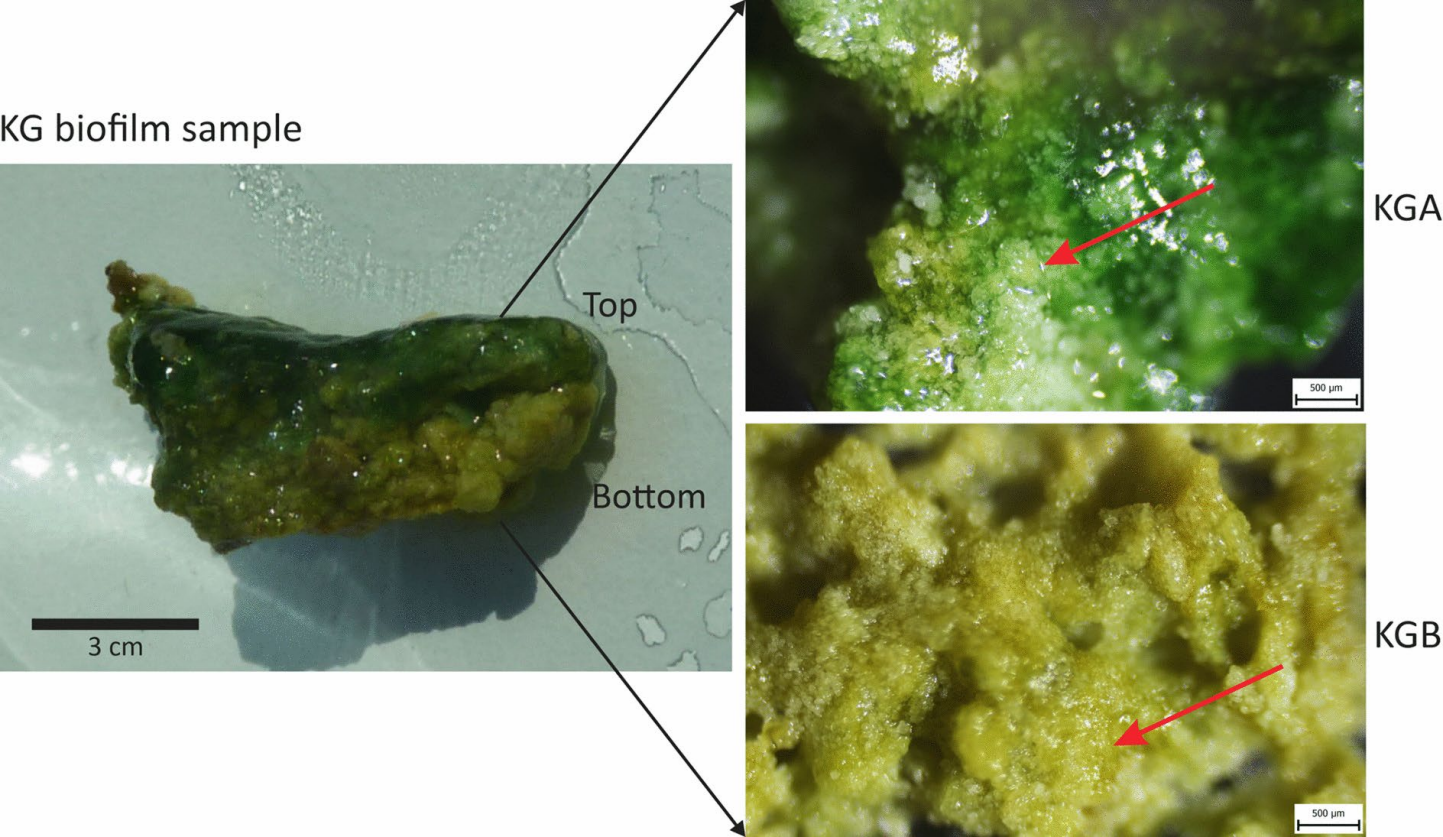
The Köröm thermal well green [KG] biofilm sample and its two isolated layers. Close-up photo (left) of a cross-section of the green [KG] biofilm sample. The sample shows an exterior (upper panel), greenish blue colour [KGA] and an interior (lower panel), pale green colour [KGB] layer. The red arrows mark carbonate precipitates.

We collected samples from the flat, several centimetre thick multilayered biofilms (Figs. 2, 3), which had a cohesive gelatinous structure and were easily separated from the underlying hard sediment. Lifting the samples with a shovel reveals their horizontal stratification (Fig. 2 and Supplementary Fig. S1 online). The ~ 5 cm thick KR (red) biofilm consists of five different coloured layers (from KRA to KRE samples), and the ~ 3 cm thick KG (green) biofilm contain two distinct layers (KGA and KGB samples) with unique colours (Fig. 3).

Based on stereomicroscopic images, the carbonate deposition trends of the red and green coloured biofilm samples were distinct, but no horizontal laminations occurred in the mineral proportions. In the red biofilm (KR), the mineral grains were arranged in a filamentary structure. The orientation of these filaments was perpendicular to the substrate (Fig. 2, Supplementary Figs. S1 and S2 online). Following the terminology of Dupraz et al.^38^, KR biofilm showed internal fibrous features that are found in the stromatolites, but were not themselves laminated stromatolites. In the green biofilm (KG), the mineral grains showed a dense mesoscopically irregular clotted internal texture, which is characteristic of thrombolitic microbialites. The exterior layer of the KG sample was darker green (greenish blue) than the interior, which could imply the microbial community with greenish blue pigmentation was more abundant on the surface (Fig. 3).

**Fig. 4:**
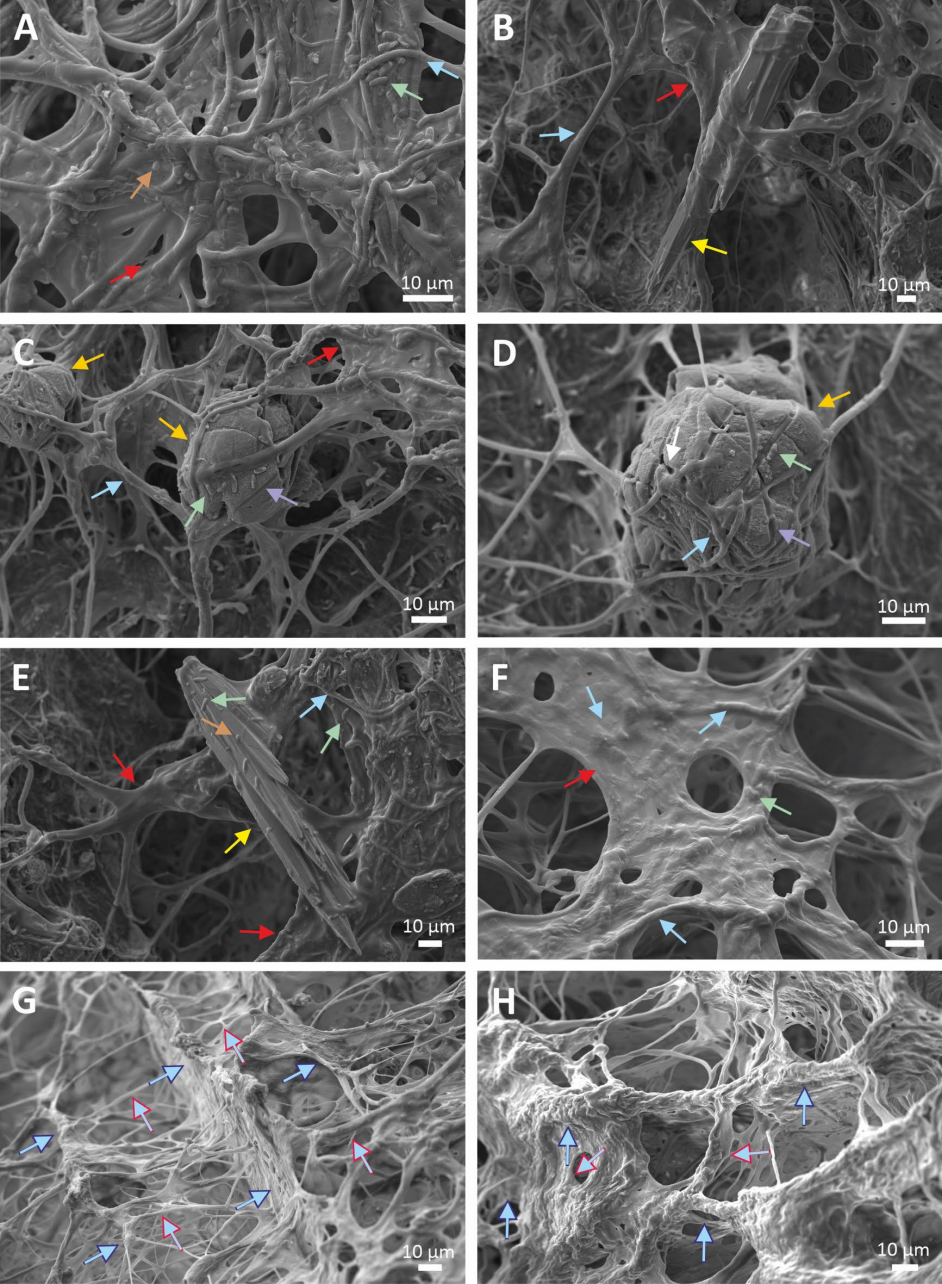
SEM micrographs of 5% glutaraldehyde-fixed and lyophilized samples of the multilayered red (KR) biofilm show the net-like texture of several type of bacterial cells embedded in a mucilaginous material with mineral particles: KRA layer (**A**-**B**), KRB layer (**C**-D), KRC layer (**E**), KRD layer (**F**) and KRE layer with ladder-like structures (**G**-**H**). (Brown arrows: cocci, green arrows: rods, blue arrows: filaments, red arrows: extracellular polymeric substances, white arrows: bacteria-shaped holes, purple arrows: bacterial molds, yellow arrows: euhedral, presumably abiotic aragonite, orange arrows: peloids, blue contour blue arrows: long longitudinal filament bundles, red contour blue arrows: shorter transverse filaments).

**Fig. 5:**
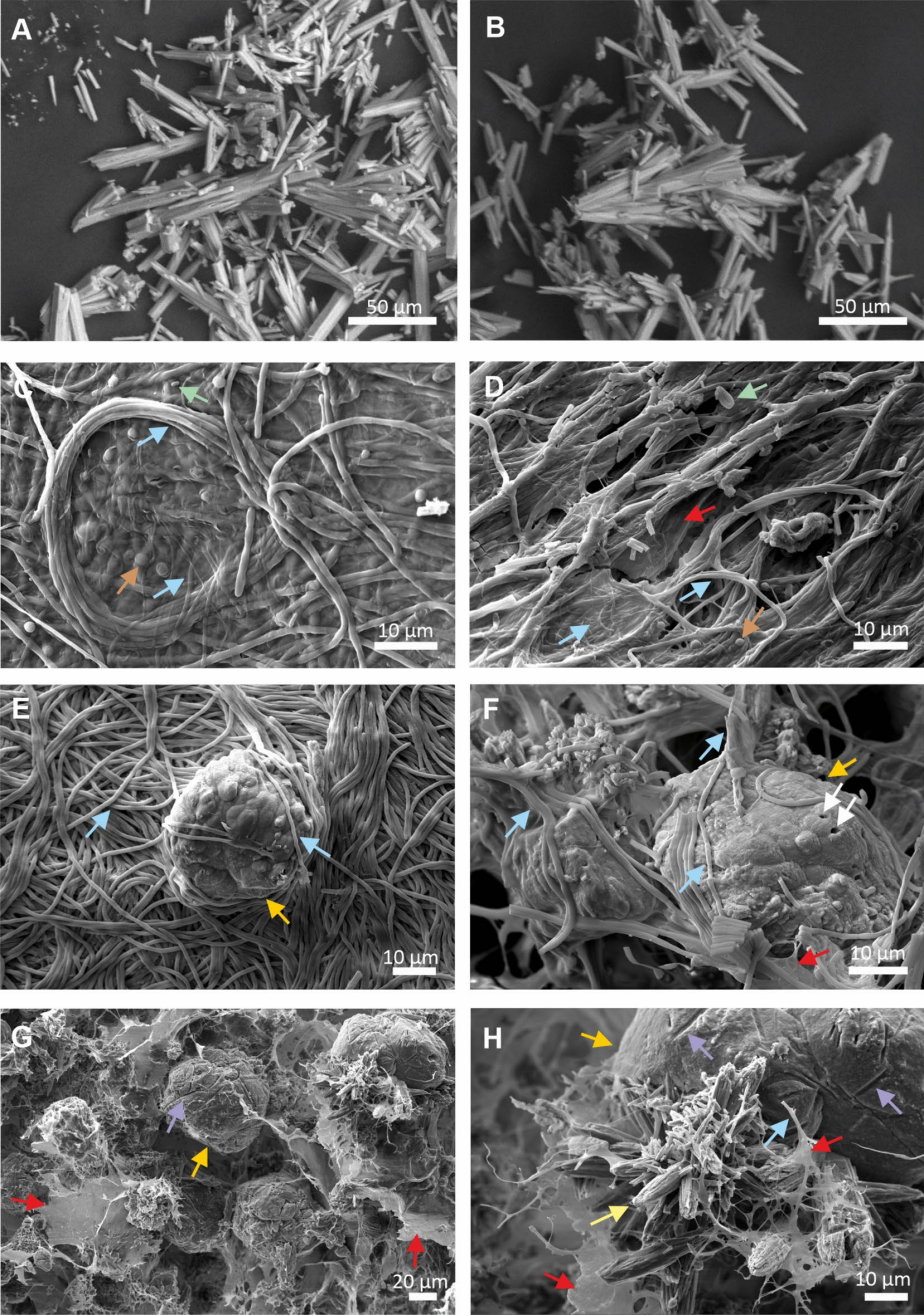
SEM micrographs of 5% glutaraldehyde-fixed and lyophilized samples of K1 precipitated carbonate without visible bacterial cells and layered green (KG) biofilm of filamentous bacteria that contain aragonite: K1 sample with abiotic aragonite (**A**-**B**), KGA layer (**C**-F) and KGB layer (**G**-**H**). (Brown arrows: cocci, green arrows: rods, blue arrows: filaments, red arrows: extracellular polymeric substances, white arrows: bacteria-shaped holes, purple arrows: bacterial molds, yellow arrows: euhedral, presumably abiotic, aragonite, orange arrows: peloids).

**Fig. 6:**
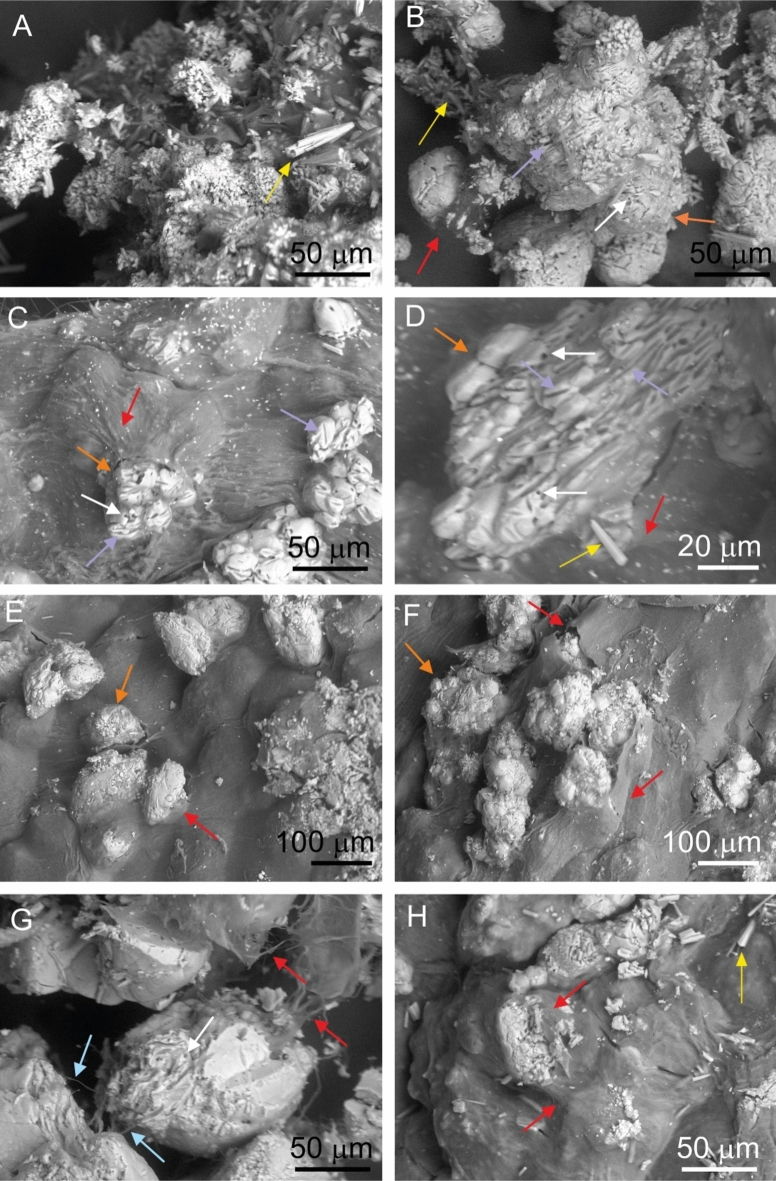
Backscattered electron images of the air-dried multilayered biofilm samples from the Köröm thermal well showing acicular and peloidal aggregates of CaCO_3_ minerals with bacterial molds embedded in the biofilms’ matrix: KGA layer (**A**), KGB layer (B), KRA layer (**C**-**D**), KRB layer (**E**), KRC layer (**F**), KRD layer (**G**), KRE layer (**H**). (Blue arrows: filaments, red arrows: extracellular polymeric substances, white arrows: bacterial-shaped holes, purple arrows: bacterial molds, yellow arrows: euhedral, presumably abiotic aragonite, orange arrows: peloids.)

The SEM images of the multilayered biofilms revealed numerous cocci, rods and bundles of filamentous structures in the size range of prokaryotes embedded in the mucilaginous material (Figs. 4, 5). The net-like texture was attributed to filamentous bacteria, resembling structures with a “ladder-like” arrangement (long longitudinal filament bundles connected by shorter transverse filaments) that was clearly detectable mainly in the red biofilm samples (e.g. Figure 4C, G-H). In addition to the mucilaginous EPS, the “ladder-like” interwoven structures helped to maintain the shape of a biofilm several centimetres in diameter. However, filamentous microbes were observed more densely adherent to each other in the uppermost layers of the biofilms than inside the biofilm (Fig. 4A, Fig. 5C-E). Each layer of both biofilm samples contained mainly mineral peloids of spherical/oval morphology in various sizes (20–60 µm) in close proximity to abundant bacterial cells and filaments (e.g. Figure 4C-D, Fig. 5E-F). The peloids formed aggregates reaching up to 250 μm in diameter (e.g. Supplementary Figs. S1C-E, S2 online, Fig. 6), which thus enabled their visibility by naked eye (e.g. Supplementary Fig. S1 online). Large numbers of bacteria, bacterial imprints and bacteria-shaped holes were found on mineral surfaces (Fig. 4C-D, Fig. 6C-D, G). Minerals precipitated in the biofilms were bright on backscattered electron (BSE) images due to their higher atomic number (Ca^2+^) compared to the low atomic number-dominated (C, H, O, N) (Fig. 6A-H) remnants of the microbes-containing EPS (e.g. Figure 6C). Figures 6C and E–G clearly showed that the bright-colour mineral units are connected by bundles of filaments. The distinctive needle-like, acicular aragonite crystals abundantly occurred in the K1 sample collected in the immediate vicinity of the well (Fig. 5A-B). Euhedral crystals are indicative for inorganic carbonate precipitation^39^ and they were only rarely observed in the aragonite-containing KR and KG biofilm samples (e.g., Fig. 4B, 4E and Fig. 5H).

The micro-XRD measurements of the carbonate precipitates and biofilm samples indicated two distinct CaCO_3_ polymorphs (Supplementary Table S2 online). Aragonite dominated the carbonate precipitates of K1, K3 and K4, whereas K2 contained calcite. The XRD results were supported by SEM images (Supplementary Fig. S3 online). The difference between aragonite and calcite was even visually detectable. The calcite deposits formed a rather hard, very compact and difficult to extract layer. In comparison, the aragonite deposits were lighter in colour and crumbly in texture. The coloured layers of the red biofilm (KR) consisted of calcite, whereas the green biofilm (KG) contained aragonite in various proportions, but aragonite dominated in its upper layer (Supplementary Table S2 online). The TEM investigation of the carbonate grain of the KRD sample revealed low Mg-containing (~ 3 mol%) nanocrystalline calcite (Supplementary Fig. S4 online).

### The taxonomic diversity of microbial communities

Well water samples (KW1 and KW2 parallels), carbonate precipitates (K1, K2, K3, K4) and different-coloured layers of the two separate adjacent biofilms (KGA, KGB, KRA, KRB, KRC, KRD, KRE) were subjected for molecular biological studies. After the bioinformatic analysis, 432 314 bacterial and 1 023 archaeal high quality sequences were obtained from the 13 samples. The relatively low number of archaeal sequences provided only a brief overview of the archaeal community. As some overrepresented taxa can easily bias the results when the number of archaeal sequences is low, the diversity indices for Archaea were ignored. For the bacterial sequences, the Good’s coverage indicated that the sequencing was representative as the coverage value exceeded 99% in each sample (Supplementary Table S3 online). The number of observed operational taxonomic units (OTUs) showed that the water (KW1, KW2) and the carbonate precipitate samples closest to the well (K1, K2) had lower species richness than the multilayer biofilm samples (KG and KR). The diversity indices indicated that the KGA (greenish blue) and the KRE (greyish green) layers as well as K4 precipitate sample had the most diverse bacterial community.

**Fig. 7:**
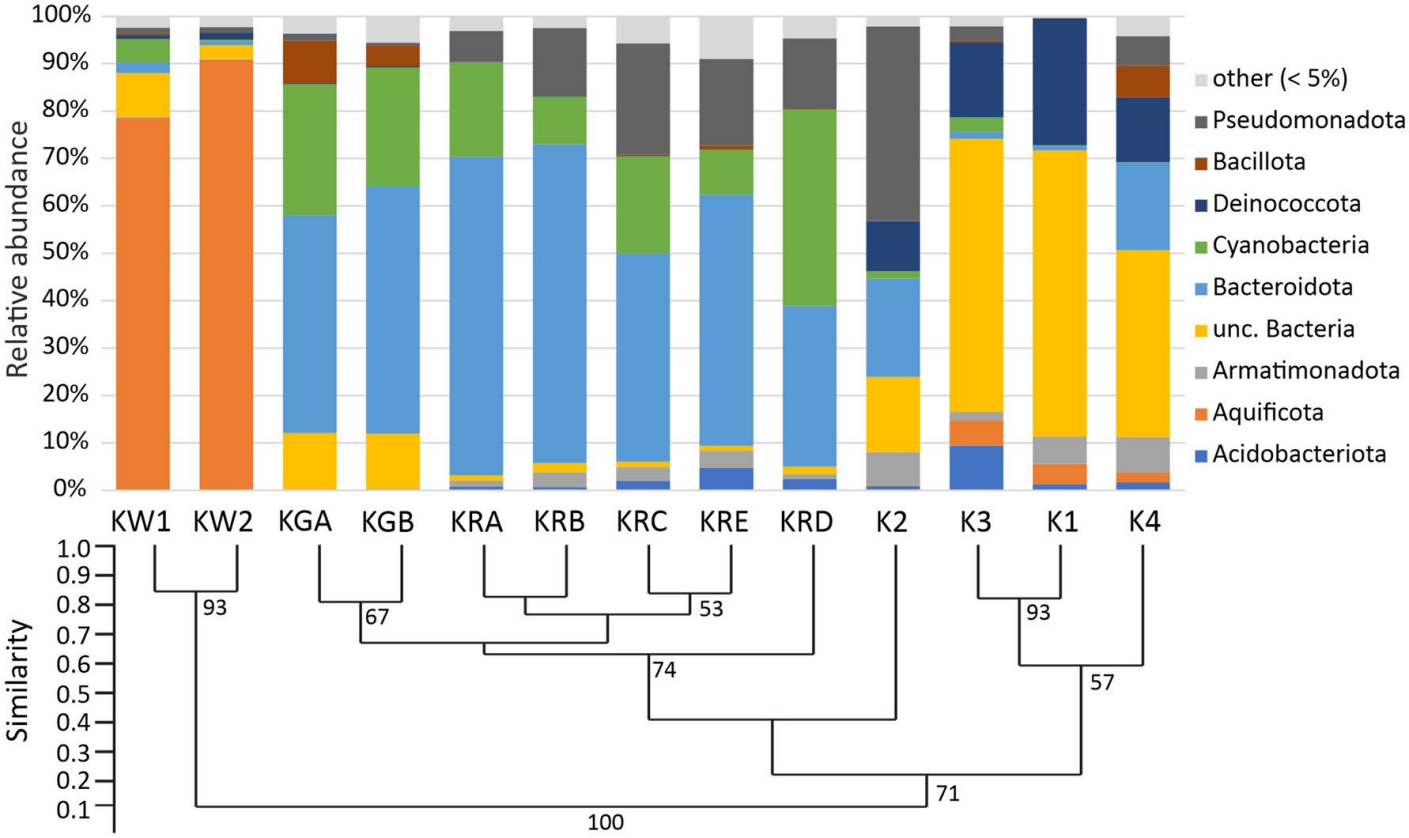
The phylum level bacterial taxonomic composition of the various Köröm thermal well samples. The UPGMA similarity analysis was conducted using the Bray–Curtis method and 500 bootstrap values. Phyla with < 5% relative abundance are classified as “other”. Water samples: KW1-KW2; green biofilm layers: KGA-KGB; red biofilm layers: KRA-KRE; carbonate precipitates: K1-K4; unclassified: unc.

**Fig. 8:**
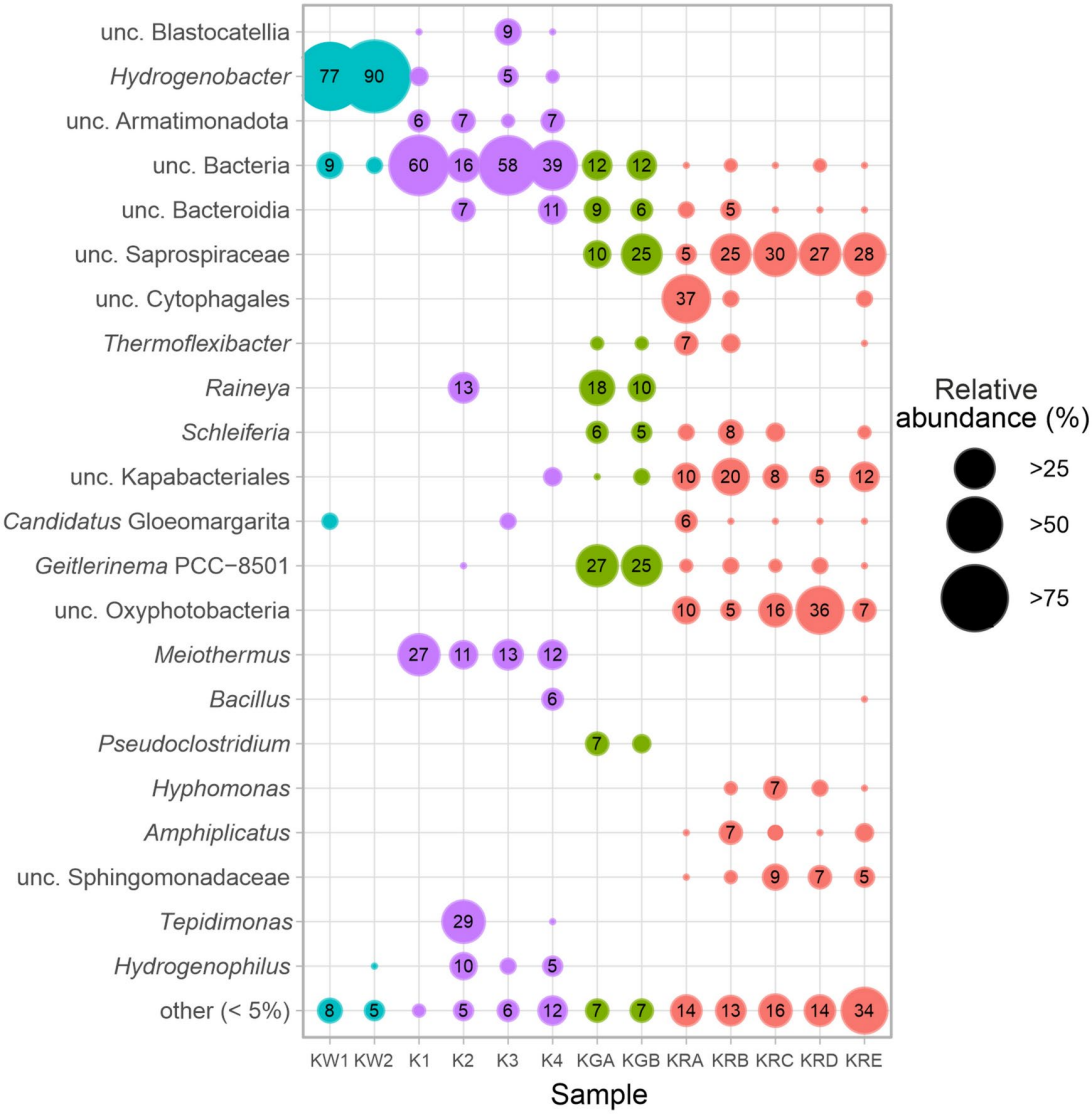
Bubble plot of the evaluation of the genus level bacterial taxonomic diversity of the Köröm thermal well samples. Water samples: KW1-KW2; green biofilm layers: KGA-KGB; red biofilm layers: KRA-KRE; carbonate precipitates: K1-K4; uncultured: unc.

The phylum and the genus level identification revealed significant differences among the different sample types (Fig. 7, 8, Supplementary Table S4 online). According to the unweighted pair group method of arithmetic averages (UPGMA) analysis, the similar sample types were colonized mainly by the same phyla but with varying relative abundance values. In the two water samples (KW1, KW2), the phylum Aquificota (*Hydrogenobacter*) had absolute (higher than 70%) relative abundance (Fig. 7, 8).

In the precipitate samples (K1-K4), the relative abundance of unclassified Bacteria (16–60%), phyla Pseudomonadota (synonym Proteobacteria) (0.1–41%) and Deinococcota (10–27%) was high (Fig. 7). The genus *Hydrogenophilus* (0.4–10%) (Pseudomonadota) was more detectable at sampling points away from the well (K2-K4; Fig. 7, 8). Members of the unclassified Bacteria were dominant in almost all precipitates in the drainage channel (Fig. 7). The only exception was the sample K2, where the genera *Tepidimonas* (29%; Pseudomonadota), *Raineya* (13%; Bacteroidota) and *Meiothermus* (11%; Deinococcota) were the three most abundant groups along with unc. Bacteria (15%) (Fig. 7, 8). The bacterial community of the carbonate precipitates showed a large variation among samples.

In the red biofilm sample (KR), the phyla Cyanobacteria (10–41%), Pseudomonadota (7–23%) and Bacteroidota (34–67%) were dominant in each layer (Fig. 7). From the phylum Bacteroidota, the following taxonomic groups were prominent in all the observed layers: class uncultured Kapabacteriales (5–20%), uncultured representatives of order Cytophagales (0.2–37%) and family Saprospiraceae (4–30%). From the phylum Cyanobacteria, class Oxyphotobacteria (5–36%) were detected in each sample of the red biofilm layer (Fig. 8). The relative abundance of groups from lower taxonomic levels showed high variability among the biofilm layers. The order unc. Cytophagales had peak abundance (37%) in the top KRA layer. In the lower biofilm layers, the class unc. Oxyphotobacteria (e.g. 36% in the KRD) and the family unc. Saprospiraceae (25–30% in the KRB-KRE) had increased abundance, whereas the presence of the order Cytophagales was moderate (0.1–2.8% in the KRB-KRE).

In the green biofilm sample (KG), the taxonomic composition was similar to the red biofilm sample only at the phylum level. The phyla Cyanobacteria and Bacteroidota were permanent members of both biofilm layers. The sole frequent representative of the Cyanobacteria phylum was the genus *Geitlerinema* (25–27%), which was the most abundant taxon in both green biofilm layers. From the phylum Bacteroidota, the genus *Raineya* (10–17.5%) and the family Saprospiraceae (10–25%) were present in high quantities. Members of unclassified Bacteria (12%) were also more abundant in the green biofilm than in the red microbial mat.

The archaeal composition consisted of three different phyla: Crenarchaeota, Halobacterota and Nanoarchaeota (Supplementary Table S5 online). Almost half of the archaeal sequences originated from the K3 precipitate sample. The most abundant genera were *Nitrosarchaeum* (1–100%)*, Pyrobaculum* (2–74%), *Candidatus* Nitrosocaldus (1–98%) and *Thermofilum* (21–25%) in the water and the carbonate precipitate samples. *Candidatus* Nitrosotenuis (63–88%) was the most abundant taxon in all layers of the red biofilm.

## Discussion

### The architecture of the biofilms

The Köröm well environment represents a unique habitat for microorganisms, especially thermophilic microbes below the hyperthermophile threshold (80 °C)^40^. Away from the well, the water temperature and the conductivity gradually decreases and the pH increases based on Egerszalók thermal well analogy. The water contains elevated amount of Na^+^ (> 330 mg/L). The decreasing water temperature and turbulence favour the precipitation of carbonate minerals, which are enhanced by the dynamic CO_2_ degassing at the water–air interface, increasing pH and microbial activity, as described by Mors et al.^41^. In contrast to microbialites reported by^42–44^, the biofilms contain no silica and sulphate, only calcite and aragonite.

The morphology of carbonates in the biofilm and biofilm-free samples differs significantly. Abiotic, needle-like aragonite abundantly precipitate along the high-temperature (79.2–61.4 °C), bicarbonate-rich karst water drainage in the biofilm-free samples (K1, K3 and K4, Supplementary Table S2 and Fig. S3 online, Fig. 5A-B). In contrast, the carbonate peloids with a spherical/oval morphology, which are characteristic biotic feature of modern and ancient microbialites, are abundant in the biofilm matrices (e.g. Figure 4C, Fig. 5G, Fig. 6). Their unique morphology is influenced by the metabolic activity and cell surface formulas of the microbes, as described in numerous literatures^45–47^. The needle-like, acicular aragonite crystals, present in small amounts in the biofilm samples (e.g. Figure 4B, Fig. 5H), are euhedral, presumably inorganic precipitates from water drift.

Microscopic observations show a diverse microbial community in the biofilms, with EPS-producing filamentous bacteria forming the structural basis in both red and green biofilms. (Figs. 4, 5). Close interactions are evident as filamentous bacteria interwove around the peloids, engraving bacterial molds and holes into the mineral surfaces. (Figs. 4C-D, 5F, 6). They are supposedly formed, when the crystals precipitated near cells, which then lyse^45^. The morphological features observed in our high-resolution electron microscope images of peloids closely resemble those of fossil coccoids, filamentous microbes, micropores, and organic-mineral associations for Mesoproterozoic thrombolites^48,49^. Microbial activity leaves distinct morphological and chemical signatures in minerals, as seen in preserved “fossil microbes” consisting of bacterial molds and peloids interwoven with bacterial filaments. Our study demonstrate that microbial remnants and their molds may be preserved both on the surface and within ancient peloids.

The living red biofilm (KR) exhibits internal fibrous features characteristic of stromatolites (Fig. 2, Supplementary Figs. S1, S2 online), with highly ordered bacterial filaments (Fig. 4). The interconnection of millions of bacteria creates a three-dimensional network structure of "ladder-like" elements, characteristic of large microbial biofilms^50,51^. Within this "ladder-like" structure, the peloids appear to be stabilized by longitudinal bundles of prokaryotic filaments (Fig. 4). Consequently, the precipitated minerals form long strands perpendicular to the substrate, resembling a string of pearls. Cross-section of the red biofilm samples—whether fresh, hydrated, or dried—clearly show the precipitated mineral chains consistently traverse the differently coloured layers (KRA-KRE) (Supplementary Figs. S1, S2 online). This vertically oriented microstructure resembles stromatolites with fibrous fabrics perpendicular to the surface^52^ and known as microdigitate stromatolites (MDS)^53–55^. Active biofilms from the Köröm well share similarities with fossilized MDS, including vertical fibers decorated with micropeloids and nanoglobules^53^. Such fibers, dating back to the Mesoproteozoic Era in China^52,53^ and the Archeozoic Eon in Australia^56^, were reported from multiple sites. Raman spectroscopy revealed the fossilized fibers of microdigitates were rich in organic carbon and trapped transition metals in the proximity of the digitations^56^. The organic carbon content of the vertical fibers suggested biotic origin, and the microdigitates likely represented fossilized bacterial filaments coated with mineralized micropeloids^53,56^ decorated with micro-scale peloids, consistent with precipitated minerals adhering to the outer surface of these filament^53^.

The unique filamentous carbonate grain pattern of the red biofilm (Fig. 2, Supplementary Figs. S1, S2 online) has not previously been described in morphological studies of active microbialites. The tight arrangement of the bacterial filaments in the red biofilm could have formed in the same way as the ancient MDS. The “ladder-like” arrangement of the prokaryotic filaments probably provided the structural basis for the radial fibrous fabrics observed in the fossilized microdigitate stromatolites. The horizontal lamination in the fossilized records, which led to the overall stromatolitic mesostructure, was probably the outcome of temperature dependent seasonal variations, which had a prominent effect on the microbial biomass production^53^. However, in both the fossilized and the active microbialites, the vertical stratification might be achieved by in situ mineralisation along the bacterial filaments perpendicular to the bottom. Carbonate grains arranging in a vertically laminated pattern along filamentous microbes within very thick (several centimetres) biofilms provide example that the filamentous organisms such as cyanobacteria (e.g. Oxyphotobacteria, *Geitlerinema,* Supplementary Note 1 fifth paragraph) may also play an important role in the formation of fibrous stromatolites by creating biofilms with an ordered network structure. The high degree of similarity between the fossilized microdigitate stromatolites and the micro-, and mesostructure of the Köröm well red biofilm suggests that the formation of the ordered network structures could have started in a similar way in the geologic past as it does today.

Both layers of the green biofilm (KG) show the characteristics of clot-like mesofabrics, formed by calcium carbonate minerals as thrombolite (Fig. 3). A large number of clots ~ 250 µm in diameter (Fig. 6) fill the biofilm to its full thickness. The propagation of microbialites with thrombolytic mesostructures is common in marine tidal zones^57,58^, hypersaline waters^59^ and continental hot springs^60^. Thrombolite formation tends to be coupled with the life processes of heterotrophic eukaryotes, but water level fluctuations and associated turbidity variability also contribute to their development^38,57^. However, the grazing pressure and perturbance caused by eukaryotes, were absent in the Köröm thermal well due to the high water temperature^61^, so the sharp change in the meso- and microstructure of the two neighbouring biofilms may be explained by the variation of the prokaryotic composition (Fig. 8, Supplementary Table S4 and Note 1 online). However, the green biofilm may have been subjected to greater mechanical stress than the red biofilm, as it was closer to the main flow path, formed in the splash zone, and thus probably exposed to higher water temperatures than the more distant red biofilm sample. Thus, in relation to the substrate, the vertical fibrous fabrics characteristic of red biofilms could not form in the green biofilm, but instead the clotted fabric characteristic of thrombolites appeared.

Although a significant proportion of taxa could not be identified, the known metabolic properties of the most closely related species suggest that mainly oxygen-producing thermophilic phototrophs, aerobic chemolithotrophs (hydrogen-oxidation, sulphur-oxidation) and heterotrophs inhabited the interconnected networks of biofilms, the thermal water, and the carbonate precipitates. Both the metabolic activity of the bacterial communities and the structural properties of the biofilm were essential for the mineral precipitation. Several SEM images taken from the biofilms indicated the close association between the bacterial filaments and the precipitated mineral grains. The EPS secreted by microbes, which causes the gelatinous consistency of biofilms and the cellular surfaces (e.g. cell wall, S-layer) contributed to carbonate precipitation in the microbial mats, by providing nucleation sites for the crystal nuclei (“crystal seed”) formation, which were necessary for further mineralisation processes^6,7^. Several identified bacteria have representatives known for their involvement in carbonate mineral precipitation through their strong urease^62^ or photosynthetic activity^63^ (see also Supplementary Note 1 fifth paragraph). Urease activity increases the pH of their microenvironment through ammonia production^62^, and the photosynthesis absorbs carbonate ions from the solution and shifts the equilibrium to carbonate ions^63^. Active calcite precipitation by heterotrophic bacteria in calcium-rich environments is a consequence of a detoxification strategy. The incorporation of excess metal ions into carbonates occurs extracellularly to maintain cellular calcium homeostasis^64^. For instance, some members of the genera *Bacillus* (Bacillota), *Tepidimonas* (Pseudomonadota), *Geitlerinema* and *Gloeomargarita* (Cyanobacteria) are known calcium carbonate precipitating bacteria^65–67^. These bacteria may have contributed to the formation of peloids within the biofilm with characteristic morphologies similar to those previously described in fossil microbialites.

Despite the similarities in the environmental conditions (pH, temperature, high dissolved HCO_3_^-^ content) and morphological characteristics of biofilms (Supplementary Note 2 online) from hot springs in Yellowstone National Park (USA)^55,69^, Central Italy^20^ and Baikal rift zone (Russia)^1^, the microbial communities and mineral content of the microbial mats were not uniform. The high arsenic content in the thermal water and the lack of anoxic layers within the biofilms could also contribute to the differences in the bacterial communities. The potential arsenate-reducing archaea were detected in the water and the drainage channel of Köröm (Supplementary Note 1 online). The oxygen-producing photosynthesis of Cyanobacteria detected in the lower layers of the Köröm well biofilm samples did not allow the formation of a permanent anoxic microenvironments, usually characteristic of microbialites^6,7^, and explains the low abundance of archaeal sequences in our samples as compared to literature data^20^ (Supplementary Table S5 online).

The comparison of modern and fossil microbialites is challenging due to the differences in their environment, formation processes and conservation status. Several studies have shown that hot spring carbonates do not contain fossil microbes/EPS, in contrast to siliceous thermal spring deposits^70–72^. Microbialites, such as stromatolites and thrombolites and various microbially induced sedimentary structures were well-preserved in the Mesoproterozoic Wumishan Formation, deposited in the peritidal or the subtidal environments of anoxic and carbonate-supersaturated seawaters^46,48,49,53,54,73^. Despite the environmental differences, the ancient Mesoproterozoic microbialites likely formed through bacterial mineralisation similar to modern Köröm thermal well biofilms. Without eukaryotes, filamentous cyanobacteria and other prokaryotes likely played a key role in promoting local carbonate precipitation.

The geomicrobiological study of Köröm well suggests that the red biofilm represents a developmental stage in the development of MDS, based on the vertically elongated, regular network of microbial filaments observed in close association with peloidal fibrous fabrics. The appearance of peloids interwoven with bacterial filaments and bacterial-sized holes on the surface in both the red and green biofilms provide a clear explanation for understanding bacterial fossils (filaments, molds; EPS and closely associated nanoglobules) described in the silicified microbialies of the Wumishan Formation.

## Methods

### Sampling

Microbial biofilms, carbonate precipitates and water samples were collected on 11 August, 2020. Water temperature and electric conductivity were measured in situ using a WTW ProfiLine pH/Cond 3320 device (Supplementary Table S1 online). Detailed physico-chemical analyses of water samples K1 and K5 were performed in the laboratory using the methods described in the article by Kondor et al.^74^.

For molecular biological studies, one L water samples (KW1 and KW2 parallel) were collected in sterile glass bottles at the sampling points from the water outlet. Carbonate precipitates were obtained under running spring water with a sterilized spatula from four distinct sites of the well (from samples K1 to K4), where thick mineral depositions were observed. Two separate adjacent biofilms, 5–6 m from the wellhead and out of the main flow path, were collected with an ethanol-sterilized shovel and placed in sterile glass bottle (Fig. 1). All collected samples were kept at 4 °C until laboratory processing. In the laboratory, the biofilm samples were cut to approximately the same size using ethanol-sterilised scissors and the layers were separated using sterilised needles and tweezers (Figs. 2, 3).

### Environmental DNA extraction

DNA isolation from all sample types was conducted by the DNeasy PowerSoil® Kit (Qiagen). The water samples were filtered through nitrocellulose membrane filters (MF-Millipore™) (0.2 µm pore diameter) using a vacuum pump to collect the microbial biomass. The filters were sliced up to smaller pieces and loaded onto the PowerBead Tube of the DNA isolation kit. Equal amounts of the biofilm samples and 0.2 g of mineralized precipitates were homogenized in a sterile mortar, then the biomass was loaded into the DNeasy PowerSoil® Kit (Qiagen) PowerBead Tube. The DNA isolation was performed according to the manufacturer’s descriptions, except for step 3 where a mixer mill (Mixer Mill MM301, Retsch) was used for cell disruption for 2 min at 30 Hz. The concentration of 5 µl of the isolated DNA was measured using the Qubit dsDNA HS Assay Kit (Thermo Scientific).

### Next-generation sequencing (NGS) and bioinformatic evaluation

During the PCR process, the V3-V4 region of bacterial 16S rRNA gene-sequences were amplified using the Bacteria-specific Bact-341F and Bact-805NR^75^ and the Archaea-specific Arch-519F and Arch-855R^76^ primer pairs. In both cases, the first section of the primers 5’ end contained the Fluidigm CS1 and CS2 tags. The Bacteria-specific PCR mixture contained 9.9 µl DEPC-treated water, 4 µl 5 × Phusion II HF buffer (Thermo Scientific), 4 µl dNTP mixture (1 mM) (Thermo Scientific), 0.4 µl Bovine Serum Albumin (20 mg/ml) (Thermo Scientific), 0.25–0.25 µl from both Bacteria-specific primers (40 µM), 0.2 µl Phusion II Hot-Start HF polymerase (2 U/µl) (Thermo Scientific) and 1 µl template. The temperature protocol was the following: 3 min initial denaturation at 98 °C; 25 cycles of denaturation at 95 °C for 10 s, primer annealing at 55 °C for 30 s and elongation at 72 °C for 30 s; PCR was concluded by a single final extension step at 72 °C for 5 min. The amplification of the archaeal 16S rRNA gene region required a distinct PCR mixture, which was prepared from 9.8 µl DEPC-treated water, 4 µl 5 × Phusion II HF buffer (Thermo Scientific), 4 µl dNTP mixture (1 mM), 0.4 µl Bovine Serum Albumin (20 mg/ml), 0.3 µl from the Archaea-specific primer pair (40 µM), 0.2 µl Phusion II Hot-Start HF polymerase (2 U/µl) and 1 µl template. Its temperature protocol was the following: 5 min initial denaturation at 98 °C; 25 cycles of denaturation at 95 °C for 10 s, primer annealing at 60 °C for 30 s and elongation at72 ^o^C for 30 s; PCR was concluded by a single final extension step at 72 °C for 5 min. All PCR were performed in triplicate for standardization (3 × 20 µl). The quality of the amplification step was assessed by 1% agarose gel electrophoresis. The gel contained 0.8 g agarose, 80 ml 1 × TBE (Tris–Borate-EDTA) buffer and 2 µl ECO Safe Nucleic Acid Staining Solution (Pacific Image Electronics). GeneRuler 100 bp DNA ladder (Thermo Fisher Scientific) was used as molecular marker. 5 µl of the PCR products were loaded onto the gel with 5 µl loading dye, consisting of 30% (v/v) glycerine and 0.25 mM bromophenol blue, and run under 100 V for 35 min. The visualization was performed by UV light. After the quantity check, the triplicates were merged in a single PCR tube and kept under -20 °C until further processing.

DNA concentrations of the amplicons were measured and normalized by Qubit dsDNA HS Assay Kit. The final DNA libraries were cleaned, quantified, barcoded and sequenced on the Illumina MiSeq platform by the Genomics Core, Research Technology Support Facility, Michigan State University (USA). Sequencing was performed on a MiSeq v2 flow cell with 500 cycles and 2 × 250 bp paired end format. The sequencing data can be accessed in the NCBI SRA database under the PRJNA836382 Identification Number. The sequencing data were evaluated by using the mothur v. 1.44.3 software program package^77^. The evaluation followed the guidelines of the MiSeq SOP (http://mothur.org/wiki/miseq_sop/ downloaded on 27.04.2021.)^78^. The first steps of the raw sequencing data processing were the following: quality check (deltaq = 10), exclusion of homopolymers (max. 7) and sequences originating from sequencing or amplification errors and exclusion of sequences with inadequate length (≥ 400–500 bp). The filtered sequences were fitted to the SILVA SSU NR database (Release 138)^79^. Only matches where the bootstrap confidence value exceeded 80 out of 100 iterations were kept. The UCHIME software was used to remove the artefact chimeric sequences^80^ and singleton sequences were also removed according to Kunin et al.^81^. Sequences derived from the chloroplasts, mitochondria, eukaryotes and of unknown origin were all removed. For searches in the Archaea/Bacteria domain, only sequences from the target domain were used. Sequences with at least 97% sequence identity were classified into the same operational taxonomic units (OTUs)^82^. The Shannon and the Inverse Simpson diversity indices, the Chao1 species richness and Good’s coverage were also calculated with mothur program package. The Bray–Curtis similarity index was based on the unweighted pair group method of arithmetic averages (UPGMA), the dendrogram of bacterial phyla was constructed using the PAleontological Statistics (PAST3) software^83^ with 500 iterations. Bubble plot was created using ggplot2 package^84^ in R version 4.3.0^85^ based on the relative abundances of the genera.

### Microscopy

Light microscopy images of the multilayered biofilm samples (KRA-KRE and KGA-KGB) were captured at 40–1000 × magnification using a Canon camera attached to the microscope and they were processed with QuickPHOTO CAMERA 3.1 software.

The protocol of Anda et al.^86^ was applied to prepare the glutaraldehyde fixed samples for SEM observation. SEM images were acquired at 10 keV using an EVO MA 10 Zeiss SEM. Selected biofilm layers were dried at room temperature and fixed on a metal SEM sample holder using double sided conductive carbon tape. The texture and morphology of 10 areas from these non-conductive samples were investigated in low vacuum mode (100 Pa) using the backscattered-electron (BSE) detector of a JEOL JSM-IT700HR electron microscope at an accelerating voltage of 20 kV.

Bright-field transmission electron microscope (BFTEM) images, selected-area electron diffraction patterns and energy dispersive X-ray spectroscopy (EDS) data were obtained from a carbonate grain of the KRD sample, prepared following the procedure described by Enyedi et al.^45^, using a 200 keV Talos Thermo Scientific transmission electron microscope.

### XRD analysis

0.5–1 g of crystalline material was separated from the samples KRA, KRB, KRC, KRD, KRE, KGA, KGB, K1, K2, K3 and K4 samples and were dried at room temperature. Their mineral composition was measured using a RIGAKU D/MAX RAPID II diffractometer following the method described by Kovács and colleagues^87^.

## Supplementary Information

Supplementary Information.

## Data Availability

The raw 16S rRNA gene sequences generated in the present study were deposited in the Sequence Read Archive (SRA) database (https://submit.ncbi.nlm.nih.gov/subs/sra/) under accession number PRJNA836382.
