# Supplementary material for: Actively forming microbial mats provide insight into the development of microdigitate stromatolites
Source: Sci Rep. 2025 Feb 14;15:5497. doi: 10.1038/s41598-025-90175-0 (PMC11829031; doi:10.1038/s41598-025-90175-0)
Supplement: Supplementary file 1 — Supplementary Information. [file 41598_2025_90175_MOESM1_ESM.pdf]

Actively forming microbial mats provide insight into the development of microdigitate stromatolites

Judit Makk[1], Ábel Csongor Németh[1,2,3], Erika Tóth[1], Péter Németh[4,5], Ivett Kovács[4], Attila Demény[4], György Sipos[6], Andrea K. Borsodi[1,7], Nóra Tünde Lange-Enyedi[1,4,6]

[1]Department of Microbiology, ELTE Eötvös Loránd University, Pázmány P. sétány 1/C, H-1117 Budapest, Hungary

[2]Department of Public Health Laboratories, National Public Health and Pharmaceutical Center, Albert Flórián Street 2-6., H-1097 Budapest, Hungary

[3]Doctoral School of Environmental Sciences, ELTE Eötvös Loránd University, Pázmány Péter Street 2., H-1117 Budapest, Hungary

[4]Institute for Geological and Geochemical Research, HUN-REN Research Centre for Astronomy and Earth Sciences, Budaörsi út 45, H-1112 Budapest, Hungary

[5]Research Institute of Biomolecular and Chemical Engineering, Nanolab, University of Pannonia, Egyetem út 10, H-8200, Veszprém, Hungary

[6]Functional Genomics and Bioinformatics Group, Faculty of Forestry, University of Sopron, Bajcsy-Zsilinszky út 4, H-9400, Sopron, Hungary

[7]Institute of Aquatic Ecology, HUN-REN Centre for Ecological Research, Karolina út 29, H-1113 Budapest, Hungary

Supplementary Table S1. Water parameters measured *in situ* at Köröm thermal well.

Sample sign	*K1*	*K2*	*K3*	*K4*	*K5*
Distance from the well (m)	0	2	7	18	20
Water temperature (^{o}C)	79.2	-	63.7	62.3	61.4
Conductivity (µS/cm)	1554	-	1524	1517	1514

Supplementary Table S2. Mineral composition of the Köröm thermal well samples measured by micro-XRD.

Sample sign	Sample type	Result of micro-XRD analysis
K1	carbonate precipitate	aragonite
K2	carbonate precipitate	calcite
K3	carbonate precipitate	aragonite
K4	carbonate precipitate	aragonite
KGA	green biofilm	30% calcite, 70% aragonite
KGB	green biofilm	55% calcite, 45% aragonite
KRA	red biofilm	calcite
KRB	red biofilm	calcite, trace amount of aragonite
KRC	red biofilm	calcite
KRD	red biofilm	calcite
KRE	red biofilm	calcite

Supplementary Table S3. Diversity indices and Good's coverage values calculated by the mothur program package based on a subsample of 23 525 sequences. Carbonate precipitates: K1-K4; red biofilm layers: KRA-KRE; green biofilm layers: KGA-KGB; water samples: KW1-KW2.

**Sample sign**	**Number of reads**	**Number of OTUs**	**Good's coverage (%)**	**Chao1**	**Shannon**	**Inverse Simpson's (1/D)**
K1	37 356	81	99.95%	87.0	1.83	4.77
K2	23 525	161	99.99%	161	2.45	6.83
K3	42 202	483	99.49%	542	2.68	5.53
K4	31 605	228	99.84%	238	2.77	7.65
KGA	43 909	565	99.39%	638	3.26	9.75
KGB	30 704	392	99.81%	403	2.91	7.58
KRA	29 691	411	99.80%	421	3.13	8.65
KRB	35 405	515	99.62%	545	3.15	8.55
KRC	32 619	332	99.79%	347	2.83	7.24
KRD	24 228	307	99.97%	307	2.46	4.74
KRE	29 899	480	99.76%	493	3.54	10.0
KW1	34 292	269	99.79%	287	1.39	1.73
KW2	36 879	68	99.96%	72.2	0.57	1.22

Supplementary Table S4. Relative abundance of identified bacterial sequences at the phylum and the genus level in the Köröm thermal well (Genera having >5% relative abundance in a sample are marked out in bold. Abbreviations: KW1-2: water samples; KGA-B: green biofilm layers; KRA-E: red biofilm layers; K1-4: carbonate precipitates; unc.: unclassified.)

Taxon	KW1	KW2	K1	K2	K3	K4	KGA	KGB	KRA	KRB	KRC	KRD	KRE
**Acidobacteriota**	0.12	0.12	1.27	0.85	**9.48**	1.65	0.09	0.22	0.83	0.66	1.99	2.35	4.73
Paludibaculum				0.63	0.06	0.63	0.04	0.12	0.03	0.12	0.86	0.88	0.60
unc. Acidobacteriae	0.01			0.04	0.03	0.02	0.01	0.01		0.03	0.06	0.15	0.98
Bryobacter	0.01	0.01						0.03	0.78	0.45	1.00	1.28	2.29
unc. PAUC26f	0.01									0.03	0.06	0.03	0.27
unc. TSBb06				0.02									
unc. Acidobacteriota						0.01							
Blastocatella								0.01					
unc. Blastocatellaceae									0.01				0.02
unc. Blastocatellaceae	0.01	0.02						0.01		0.03		0.02	0.54
Stenotrophobacter													0.02
Tellurimicrobium	0.01												
**unc. Blastocatellia**	0.08	0.09	1.27	0.16	**9.35**	1.01	0.03	0.05					
Pyrinomonas					0.04								
unc. Subgroup 10													0.01
unc. Vicinamibacteraceae											0.01		
uncultured Vicinamibacterales									0.01				
**Actinomycetota**	0.01	0.04	0.01	0.01	0.01	0.03		0.01					
unc. IMCC26256			0.01										
Ilumatobacter		0.01				0.02							
Corynebacterium	0.01	0.01											
Mycobacterium				0.01									
uncultured Euzebyaceae						0.01							
uncultured Frankiales								0.01					
unc. Microbacteriaceae	0.01												
unc. Micrococcaceae		0.02											
Nocardioides					0.01								
**Aquificota**	**78.46**	**90.75**	4.29	0.13	**5.29**	2.06	0.03				0.02		0.04
unc. Aquificae	0.41	0.15		0.01	0.02								
unc. Aquificaceae													
unc. Aquificaceae	1.46	0.14		0.01	0.29	0.01							
**Hydrogenobacter**	**76.60**	**90.46**	4.29	0.11	4.94	2.05	0.03				0.02		0.04
unc. Aquificota					0.04								
**Armatimonadota**	0.24	0.15	**5.78**	**7.12**	1.75	**7.47**	0.10	0.07	1.25	3.14	2.92	1.09	3.63
unc. Chthonomonadales						0.22							
unc. Fimbriimonadaceae	0.11						0.01		0.03	0.03	0.08	0.01	3.19
unc. Fimbriimonadales										0.02			
unc. Fimbriimonadales	0.02								1.21	3.07	2.73	1.05	0.39
**unc. Armatimonadota**	0.12	0.15	**5.78**	**7.12**	1.75	**7.26**	0.09	0.07		0.01	0.11	0.02	0.05
**Bacteria**	**9.18**	2.89	**60.39**	**15.81**	**57.65**	**39.46**	**11.87**	**11.64**	1.14	1.94	1.16	1.55	0.97
**unc. Bacteria**	**9.18**	2.89	**60.39**	**15.81**	**57.65**	**39.46**	**11.87**	**11.64**	1.14	1.94	1.16	1.55	0.97
**Bacteroidota**	2.27	0.78	1.00	**20.77**	1.51	18.52	**45.96**	**52.21**	**67.15**	**67.30**	**43.92**	**33.88**	**53.01**
**unc. Bacteroidia**	0.02	0.02	0.01	**6.52**	0.25	**11.03**	**8.51**	**6.24**	3.08	**5.15**	1.14	0.61	1.48
unc. 37-13				0.01			0.01	0.01	0.30	0.99	0.23	0.05	0.62
Aurantisolimonas	0.01												
unc. Chitinophagaceae									0.01				

Supplementary Table S4. (continued)

Taxon	KW1	KW2	K1	K2	K3	K4	KGA	KGB	KRA	KRB	KRC	KRD	KRE
Flavisolibacter									0.01				
Flavitalea													
Terrimonas													0.02
unc. UTBCD1													0.04
unc. Chitinophagales							0.01			0.16	0.04	0.01	0.36
Haliscomenobacter									0.15	0.67	0.10		0.11
unc. Saprospiraceae								0.01		0.01			
**uncultured Saprospiraceae**	0.30	0.01		0.04	0.02		**9.71**	**24.80**	4.66	**25.06**	**30.25**	**27.25**	**28.15**
uncultured Chitinophagales									0.24	0.01			0.11
Candidatus Amoebophilus												0.01	
Belliella	0.01		0.01										
unc. Cyclobacteriaceae									0.02			0.01	0.05
Cytophaga												0.03	0.36
unc. Cytophagaceae													3.74
Sporocytophaga													0.02
**unc. Cytophagales**	0.43	0.05		0.01	0.09		0.06	0.03	**37.33**	3.10	0.16	0.21	2.88
unc. MWH-CFBk5	0.01												
Eisenibacter					0.04				0.04				0.01
**Thermoflexibacter**	0.06				0.07		2.45	1.88	**7.45**	3.55	0.37	0.06	0.66
uncultured Microscillaceae	0.01			0.54		0.04	0.01	0.01	0.01		0.01		0.04
**Raineya**	0.05	0.01		**13.23**	0.07		**17.50**	**10.31**	0.05		0.02	0.01	0.02
Flavobacterium	0.01									0.01			
unc. Flavobacteriales							0.23	0.09		0.01			
**Schleiferia**	0.40			0.01	0.21	0.01	**6.32**	**5.15**	2.81	**7.93**	3.92	0.50	1.99
unc. SM1A07	0.01				0.01				0.26	0.04		0.02	0.01
unc. AKYH767													0.13
Pedobacter												0.01	
unc. Sphingobacteriales										0.01	0.01	0.01	0.20
unc. env.OPS 17						0.02							
unc. Bacteroidota					0.01	0.01	0.16	0.27	0.68	0.89	0.17	0.15	0.16
unc. GBChlB									0.08	0.04			0.04
unc. SM1H02	0.01		0.03		0.11	0.48							
unc. SR-FBR-L83													
**unc. Kapabacteriales**	0.22	0.01		0.40	0.46	3.79	0.99	3.39	**9.97**	**19.66**	**7.51**	4.94	11.79
unc. BSV26									0.01				0.04
Candidatus Kryptonium	0.67	0.69	0.95		0.13	2.50							
unc. Kryptoniaceae	0.01				0.03	0.04							
unc. CK06-06-Mud-MAS4B-21	0.02												
unc. Rhodothermaceae									0.01				
Rhodothermus	0.01				0.01	0.60							
unc. SJA-28	0.01												
**Bdellovibrionota**	0.02								0.65	0.16	0.29	0.71	0.93
unc. Bacteriovoracaceae											0.01		
Peredibacter	0.01								0.16	0.04	0.11	0.10	0.20
Bdellovibrio	0.01								0.46	0.11	0.16	0.61	0.72
unc. 0319-6G20													0.01
unc. Oligoflexales											0.01		0.01

Supplementary Table S4. (continued)

Taxon	KW1	KW2	K1	K2	K3	K4	KGA	KGB	KRA	KRB	KRC	KRD	KRE
Silvanigrella									0.02	0.01			
**Caldatribacteriota**					0.01								
Candidatus Caldatribacterium					0.01								
**Chloroflexota**	0.09	0.04		1.24	0.93	2.59	2.35	1.75	0.74	1.22	1.67	1.21	2.36
Anaerolinea					0.11								
unc. Anaerolineaceae					0.01								
Bellilinea				0.02	0.05		0.01	0.11					0.06
Thermanaerothrix					0.04								
Caldilinea				0.31	0.52	1.61	0.01	0.06					0.04
Litorilinea	0.01												
uncultured Caldilineaceae	0.03					0.02				0.01			0.08
unc. A4b	0.04	0.04		0.49	0.09		2.29	1.56	0.52	0.90	1.11	0.75	1.92
unc. A4b							0.01				0.02	0.32	0.11
unc. SBR1031							0.01						
unc. Chloroflexi	0.01			0.01	0.02	0.02	0.03	0.01	0.22	0.28	0.21	0.05	0.02
unc. Chloroflexaceae											0.01		
Chloroflexus				0.01									
Chloronema	0.01									0.03	0.33	0.09	0.09
uncultured Roseiflexaceae										0.01			
unc. KD4-96						0.01							
OLB14				0.40	0.08	0.94		0.01				0.01	0.04
**Cyanobacteria**	4.96	0.39	0.03	1.57	2.96	0.03	**27.63**	**25.07**	**19.91**	**10.02**	**20.48**	**41.49**	**9.54**
Gloeocapsa PCC-7428				0.02									
Microcoleus PCC-7113	0.01												
unc. Cyanobacteriaceae	0.01												
Cyanobacterium PCC-7202	0.13												
Geitlerinema LD9	0.01												
Symphothece PCC-7002	0.04												
unc. Nostocaceae	0.40								0.01	0.01	0.07		0.08
unc. Cyanobacteriia	0.32	0.05	0.01	0.01	0.03		0.14	0.07	1.42	0.68	0.93	1.37	1.10
**Candidatus Gloeomargarita**	2.92	0.24	0.01	0.03	2.66				**6.13**	1.25	0.54	0.59	0.69
unc. Eurycoccales									0.03				
uncultured Eurycoccales	0.25												0.01
Limnothrix	0.01								0.43	0.20	0.06		
**Geitlerinema PCC-8501**	0.24	0.07	0.01	1.42	0.03	0.03	**27.31**	**24.82**	1.80	2.91	2.50	3.06	0.63
Leptolyngbya RV74	0.01					0.01			0.17		0.01		0.10
**uncultured Oxyphotobacteria**	0.22	0.02		0.09	0.03		0.18	0.17	**9.77**	4.78	**16.13**	**36.37**	**6.74**
uncultured Oxyphotobacteria									0.02	0.01	0.02	0.03	
Nodosilinea PCC-7104	0.08												
Synechococcus PCC-7502	0.01												
uncultured Pseudanabaenaceae		0.01			0.22					0.01		0.01	
Synechococcus PCC-6312	0.25												
unc. Obscuribacteraceae	0.01												
unc. Vampirovibrionaceae	0.03								0.12	0.16	0.19	0.05	0.11
unc. Vampirovibrionales											0.03	0.01	0.08
unc. Vampirovibrionales									0.01				
**Dadabacteriota**					0.01	0.01							

Supplementary Table S4. (continued)

Taxon	KW1	KW2	K1	K2	K3	K4	KGA	KGB	KRA	KRB	KRC	KRD	KRE
unc. Dadabacteriales					0.01	0.01							
**Deinococcota**	0.80	1.37	**26.79**	**10.59**	**15.87**	**13.76**	0.20	0.25	0.04	0.02	0.08	0.03	0.01
Truepera	0.05			0.01		0.02			0.01				
unc. Deinococci					0.04		0.01						
**Meiothermus**	0.45	0.23	**26.54**	**10.56**	**13.27**	**11.91**	0.19	0.25	0.03	0.02	0.08	0.03	0.01
unc. Thermaceae	0.03	0.01	0.01	0.02	0.42	0.04	0.01						
Thermus	0.28	1.14	0.23		2.13	1.79							
**Dependentiae**		0.01							0.01	0.01	0.02	0.02	0.10
unc. Babeliales		0.01							0.01	0.01	0.02	0.02	0.10
**Desulfobacterota**	0.58	0.80	0.01		0.02			0.01	1.30	0.03	0.02	0.01	0.11
unc. Desulfolunaceae								0.01					
unc. Desulfobacterota									0.01				
Desulfomicrobium	0.01												0.02
Bradymonadales	0.04								0.93	0.03	0.01		0.02
unc. Desulfuromonadia									0.37		0.02	0.01	0.07
Thermodesulforhabdus					0.01								
Caldimicrobium													
Geothermobacterium	0.04	0.07											
unc. Thermodesulfobacteriaceae													
Thermodesulfobacterium	0.49	0.72	0.01										
**Bacillota**	0.30	0.02	0.01	0.05	0.32	**6.65**	**9.02**	4.36			0.26	0.10	0.83
Anaerobacillus						0.02							
Anoxybacillus					0.01								
unc. Bacillaceae													
unc. Bacillaceae						0.02							
**Bacillus**	0.21	0.01			0.13	**6.12**	0.04				0.25	0.08	0.78
unc. Bacillales	0.02	0.01				0.01					0.01	0.02	0.01
Sinobaca	0.01												
unc. Planococcaceae				0.01									
Alkalicoccus						0.02							
Salipaludibacillus						0.02							
Bacilli	0.01					0.08	0.01				0.01		0.02
Exiguobacterium						0.02							
Marinilactibacillus			0.01										
Lactobacillus	0.01												
Staphylococcus	0.02												
unc. Thermoactinomycetaceae						0.29							
Cellulosibacter							0.16	0.08					
unc. Hungateiclostridiaceae					0.01		0.07	0.13					
**Pseudoclostridium**				0.03	0.02		**7.37**	3.56					0.02
unc. Clostridia						0.01	0.03	0.04					
unc. Caloramatoraceae					0.01								
unc. Caloramatoraceae													
Thermobrachium					0.10		0.01	0.01					
Clostridium sensu stricto 13						0.01							
Clostridium sensu stricto 18					0.01								
Irregularibacter					0.01								

Supplementary Table S4. (continued)

Taxon	KW1	KW2	K1	K2	K3	K4	KGA	KGB	KRA	KRB	KRC	KRD	KRE
uncultured Peptococcaceae													0.01
unc. Peptostreptococcales-Tissierellales													
Thermotalea							0.29	0.11					
unc. Thermotaleaceae					0.01		0.84	0.37					
Desulfofundulus	0.01												
unc. Desulfotomaculia		0.01											
unc. Firmicutes					0.01	0.03	0.11	0.04					
uncultured Selenomonadaceae	0.01												
uncultured Firmicutes							0.09	0.03					
**Fusobacteriota**		0.03											
Fusobacterium		0.03											
**Gemmatimonadota**												0.01	
Gemmatimonas												0.01	
**Hydrogenedentes**													
Candidatus Hydrogenedens													
**Hydrothermae**	0.92	0.86	0.29	0.01	0.01	0.04							
unc. Hydrothermae	0.92	0.86	0.29	0.01	0.01	0.04							
**MBNT15**						0.06							
unc. MBNT15						0.06							
**Myxococcota**	0.01				0.41	0.28				0.01	0.02		0.21
unc. Blfdi19										0.01			
Haliangium											0.01		0.16
unc. Polyangia					0.01								
unc. BIrii41													0.02
Phaselicystis													0.04
Sandaracinus											0.01		
unc. mle1-27	0.01				0.39	0.28							
**Nitrospirota**					0.07		0.02	0.06	0.01			0.02	0.24
Nitrospira									0.01			0.02	0.24
Thermodesulfovibrio					0.07		0.02	0.06					
**Patescibacteria**	0.63	0.48	0.03		0.07	0.23			0.01			0.04	0.40
Candidatus Falkowbacteria	0.01												
unc. Dojkabacteria					0.05	0.18							0.02
unc. Absconditabacteriales (SR1)									0.01				
Candidatus Peribacteria						0.01							
Candidatus Pacebacteria						0.01							
unc. Micronomatia													0.01
Candidatus Kaiserbacteria												0.02	0.05
Candidatus Nomurabacteria												0.02	
Candidatus Zambryskibacteria													0.11
unc. GW2011-GWA2-46-7	0.62	0.48	0.03		0.01	0.04							
unc. Parcubacteria													0.21
unc. Saccharimonadales	0.01												
**Planctomycetota**	0.11	0.02		0.12	0.48	0.40	0.02	0.01	0.29	0.93	3.28	2.48	2.57
unc. OM190													0.01
unc. Phycisphaerae											0.01		0.02
unc. CL500-3	0.03					0.05					0.01		0.01

Supplementary Table S4. (continued)

Taxon	KW1	KW2	K1	K2	K3	K4	KGA	KGB	KRA	KRB	KRC	KRD	KRE
unc. Phycisphaeraceae									0.01			0.02	0.09
unc. SM1A02	0.01								0.01	0.01	0.02		0.20
uncultured Phycisphaeraceae	0.01												0.04
Tepidisphaera													0.06
unc. Tepidisphaeraceae										0.03	0.03	0.04	0.01
unc. Tepidisphaerales										0.02	0.04	0.05	0.02
unc. WD2101 soil group	0.01				0.01				0.26	0.84	2.96	2.20	1.97
Fimbriiglobus	0.01	0.01										0.01	
Gemmata				0.05	0.46	0.32					0.01		
unc. Gemmataceae	0.02						0.02		0.01	0.04	0.21	0.17	0.06
uncultured Gemmataceae	0.02			0.05									
Pirellula				0.01									
unc. Pirellulaceae	0.01												0.01
Thermogutta		0.01						0.01					
uncultured Pirellulaceae				0.01		0.01							
Planctomicrobium						0.01							
Planctopirus													0.06
unc. Planctomycetes						0.01							
unc. Planctomycetota													
**Pseudomonadota**	1.24	1.24	0.11	**40.98**	3.09	6.15	1.46	0.60	6.57	**14.42**	**23.42**	**14.84**	**18.28**
unc. Acetobacteraceae							0.01			0.10	0.19	0.14	0.04
Rhodovarius						0.26					0.02		
Roseomonas		0.02									0.02	0.01	0.01
Rubritepida							0.02	0.03	0.02		0.04	0.07	0.02
unc. Acetobacterales					0.01							0.01	
Elioraea				0.31	0.01		0.01	0.04		0.02	0.25	0.45	0.26
unc. Alphaproteobacteria	0.03				0.02			0.01	0.23	1.03	1.19	1.17	1.04
Brevundimonas				0.01									
Caulobacter													0.01
Phenylobacterium													0.01
unc. Caulobacterales										0.13	0.14	0.01	
unc. Hyphomonadaceae	0.04									0.09	0.10	0.02	0.06
**Hyphomonas**						0.01			0.03	2.06	**7.13**	3.26	0.66
unc. UKL13-1										0.01			
**Amphiplicatus**	0.03								0.81	**7.19**	3.89	0.54	3.58
unc. Parvularculaceae									0.01	0.02			
uncultured Defluviicoccales													0.04
unc. Ferrovibrionales fa											0.01	0.01	0.06
uncultured Micavibrionales													0.01
uncultured Paracaedibacteraceae										0.01	0.02		0.01
unc. Amb-16S-1323	0.01												
unc. Beijerinckiaceae											0.01	0.02	0.01
unc. FukuN57									0.05	0.02			
Methylobacterium-Methylorubrum	0.01												
Devosia	0.01												
unc. Hyphomicrobiaceae										0.01	0.05	0.10	1.09
Hyphomicrobium													0.01

Supplementary Table S4. (continued)

Taxon	KW1	KW2	K1	K2	K3	K4	KGA	KGB	KRA	KRB	KRC	KRD	KRE
unc. Rhizobiaceae	0.01												0.02
Shinella		0.01											
Bauldia													0.01
Nordella	0.01												0.04
Phreatobacter									0.32	0.13	0.11	0.21	0.05
unc. Rhizobiales									0.03	0.02	0.01		0.03
Pannonibacter	0.01												
Pseudorhodoplanes	0.02								0.01			0.05	0.09
unc. Xanthobacteraceae	0.01	0.02											
Paracoccus	0.01		0.02	0.02	0.03								
Rhodobacter												0.02	0.27
unc. Rhodobacteraceae	0.02			0.05	0.01	0.06			0.01				0.05
Rhodobaculum	0.01												
Roseinatronobacter	0.04	0.04											
Rubribacterium	0.01									0.01	0.02		0.03
Tabrizicola	0.01												0.06
Thioclava										0.01	0.01		0.10
uncultured Rhodospirillaceae	0.01								0.01	0.02	0.01		0.14
Candidatus Megaira									0.01				0.06
unc. Rickettsiales	0.01				0.01		0.03	0.02					
unc. SM2D12	0.17	0.01			0.01	0.01			2.68	0.64	0.16	0.39	0.25
unc. DSSF69												0.01	
Polymorphobacter											0.02		0.03
Porphyrobacter	0.07								0.05	0.04	0.33	0.06	0.98
Sandaracinobacter											0.01		0.09
Sandarakinorhabdus										0.01			
**unc. Sphingomonadaceae**	0.06			0.02	0.02			0.02	0.71	2.34	**9.08**	**6.86**	**5.28**
Sphingomonas	0.01												
Sphingorhabdus													
Alishewanella	0.04												
Aliidiomarina			0.01										
Aquaspirillum	0.02												
Limnobacter		0.01											
unc. Burkholderiales	0.05	0.01		0.15	0.23	0.09	0.16	0.03		0.01			0.03
unc. Comamonadaceae	0.01			0.48	0.02		0.14		0.05	0.08	0.32	1.18	1.09
Hydrogenophaga	0.01										0.01		0.01
Polaromonas											0.01		
Rhodoferax		0.01											
Rivibacter				0.02			0.01					0.01	0.05
**Tepidimonas**	0.03	0.04	0.01	**29.19**	0.07	0.51	0.47	0.12				0.01	
Variovorax	0.01												
Gallionella						0.04							
**Hydrogenophilus**	0.02	1.02	0.04	**10.38**	2.57	**5.11**							
uncultured Hydrogenophilaceae					0.01	0.02							0.01
unc. 966-1									0.01			0.01	0.03
unc. Nitrosomonadaceae	0.01												0.01
unc. oc32									0.01	0.01			0.57

Supplementary Table S4. (continued)

Taxon	KW1	KW2	K1	K2	K3	K4	KGA	KGB	KRA	KRB	KRC	KRD	KRE
unc. Oxalobacteraceae		0.01											
Azoarcus	0.01												
Methyloversatilis									0.01	0.03		0.06	0.82
unc. Rhodocyclaceae	0.01						0.53	0.22		0.01		0.01	0.02
Sulfuritalea						0.01							
uncultured Rhodocyclaceae								0.02					
unc. AAP99					0.01								
uncultured Sutterellaceae				0.33	0.01	0.01		0.02					
Cellvibrio	0.08												
Aquicella	0.02									0.01	0.01		0.04
unc. Ga0077536									0.01				0.09
unc. Gammaproteobacteria	0.01			0.01	0.01				0.01	0.01	0.03	0.03	0.19
unc. KI89A clade									0.01				0.50
Legionella	0.05				0.01				1.29	0.09	0.04	0.01	0.13
unc. Legionellaceae	0.01								0.01			0.01	
Halomonas			0.04										
Acinetobacter	0.18	0.02											
unc. Pseudomonadaceae	0.01								0.01	0.01			
Pseudomonas	0.02	0.02			0.02								
Fontimonas	0.01												
Nevskia	0.01												
Ahniella							0.03	0.05		0.05	0.14	0.09	0.13
Aquimonas										0.01			
Rhodanobacter													
unc. Rhodanobacteraceae									0.01	0.01			
uncultured Rhodanobacteraceae	0.01								0.12	0.17			0.01
Lysobacter		0.01											
Silanimonas		0.01					0.04	0.03			0.01		
unc. Xanthomonadaceae	0.01		0.01										
uncultured Xanthomonadaceae	0.01												
unc. Xanthomonadales	0.01												
unc. Proteobacteria									0.03	0.03	0.04	0.02	0.03
**Spirochaetota**	0.01			0.04	0.02	0.03	0.55	1.84				0.02	0.46
Brevinema					0.01			0.02					
Leptospira	0.01											0.02	0.46
unc. Leptospiraceae													0.01
Turneriella				0.04		0.03	0.47	1.75					
Spirochaeta								0.02					
unc. Spirochaetaceae													
Treponema							0.07	0.05					
**Sumerlaeota**	0.01								0.04	0.10	0.34	0.09	0.02
Sumerlaea	0.01								0.04	0.10	0.34	0.09	0.02
**Verrucomicrobiota**	0.03	0.01		0.73	0.05	0.56	0.71	1.92	0.06	0.04	0.11	0.07	1.55
unc. Chlamydiales												0.01	0.09
Candidatus Udaeobacter						0.01							
unc. Chthoniobacterales	0.01			0.01			0.03	0.03					
unc. Terrimicrobiaceae							0.01						

Supplementary Table S4. (continued)

Taxon	KW1	KW2	K1	K2	K3	K4	KGA	KGB	KRA	KRB	KRC	KRD	KRE
Terrimicrobium	0.01			0.71	0.05		0.63	1.86	0.04	0.03	0.10	0.06	0.03
uncultured Methylacidiphilaceae									0.01				0.05
Lacunisphaera										0.01			0.01
unc. Verruc-01													0.04
Oikopleura													0.10
unc. Pedosphaeraceae									0.01				0.58
unc. Pedosphaeraceae						0.55							0.05
unc. Verrucomicrobiae				0.01			0.04	0.03		0.01			0.33
Prosthecobacter		0.01									0.01		0.24
unc. Verrucomicrobiaceae													0.01
**WPS-2**									0.01				
unc. WPS-2									0.01				

Supplementary Table S5. Relative abundance of identified archaeal sequences at the phylum and the genus level in the Köröm thermal well (Abbreviations: KW1-2: water samples; KGA-B: green biofilm layers; KRA-E: red biofilm layers; K1-4: carbonate precipitates; unc.: unclassified.)

Taxon	KW1	KW2	K1	K2	K3	K4	KGA	KGB	KRA	KRB	KRC	KRD	KRE
**Crenarchaeota**	100	100	100	100	100	73.33	100	100	100	100	100	100	92.86
Candidatus Nitrosocaldus		1.12		23.81	98.16	70							
Candidatus Nitrosotenuis	1.89								77.27	62.50	85.71	76.92	87.50
Nitrosarchaeum		0.56	100	28.57					4.55		14.29		0.89
Candidatus Nitrososphaera													4.46
unc. Nitrososphaeraceae						3.33	100		18.18			23.08	
Candidatus Nitrosotalea								100		37.50			
unc. Desulfurococcales	1.89												
Thermofilum	20.75	24.58											
Pyrobaculum	72.64	73.74		47.62	1.84								
unc. Thermoproteales	2.83												
**Halobacterota**						26.67							
unc. Haloferacaceae						26.67							
**Nanoarchaeota**													7.14
unc. GW2011_GWC1_47_15													7.14
**Number of sequences**	106	179	1	21	488	60	4	2	22	8	7	13	112

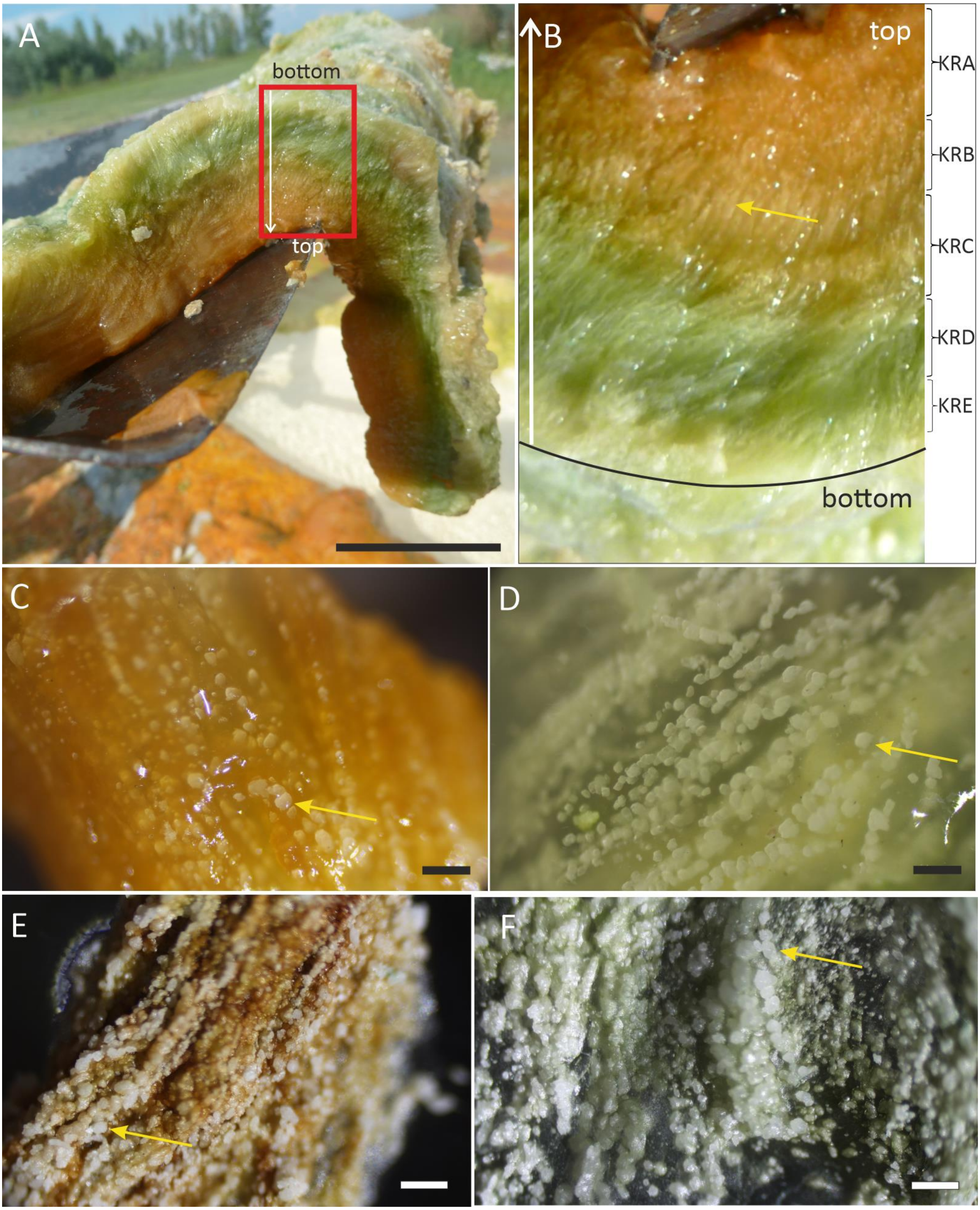

Supplementary Fig. S1. Images of living (A-D) and completely dried (E-F) KR biofilm samples. Living KR biofilm sample upside down on the shovel (A) and a section of the biofilm (B) (close view of the boxed part in A image), where continuous white fibrous/filamentous patterns indicate the orientation of carbonate precipitation (upper images, bar: 5 cm); stereomicroscopic images show isolated the living KRA (C) and KRD biofilm layer samples (D) and the completely dried out KRA (E) and KRD biofilm sample (F) (yellow arrows: carbonate precipitates; white arrows: top and botton orientations) (C, D, E and F pictures, bar: 500 µm).

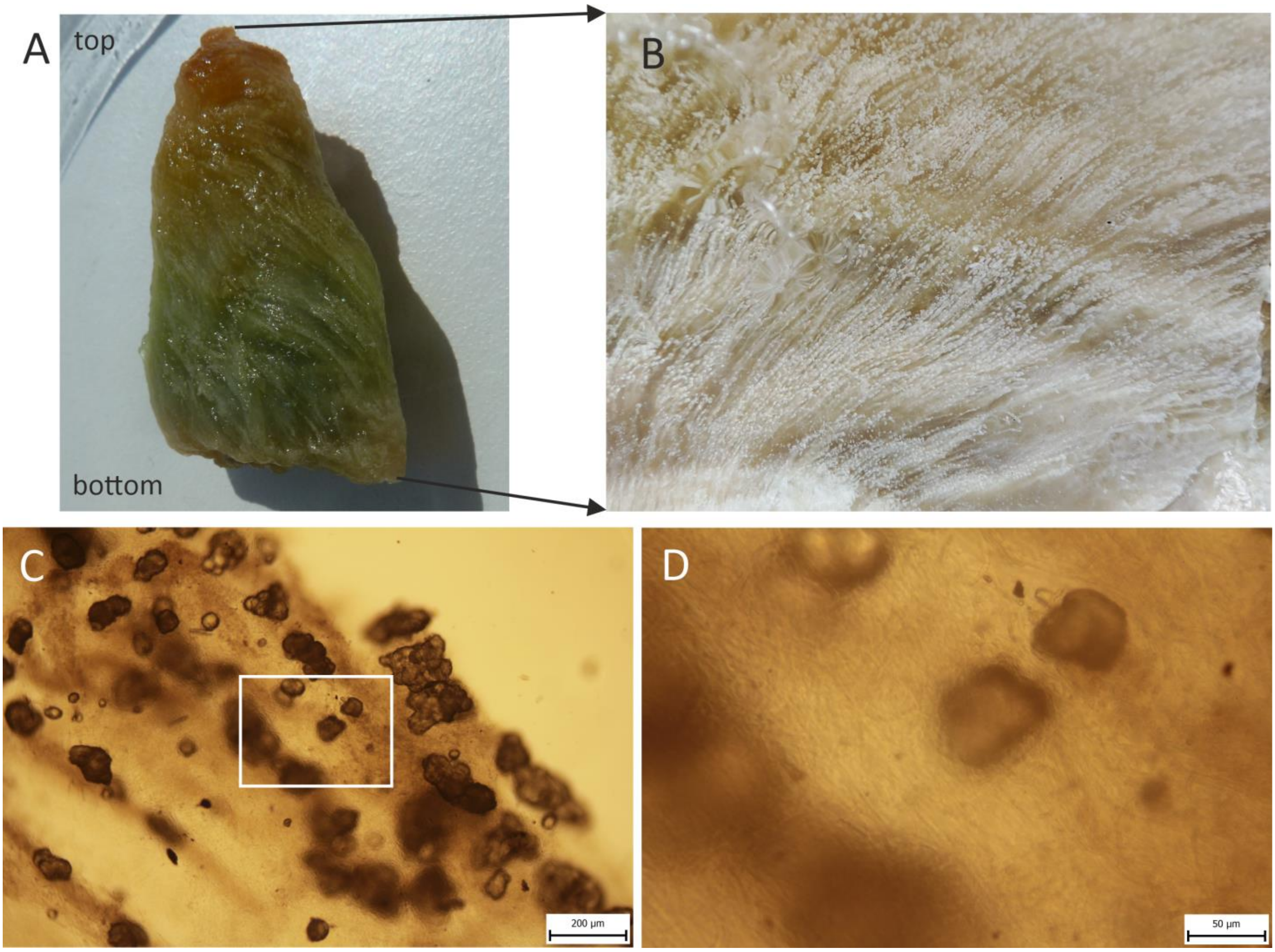

Supplementary Fig. S2. Images showing the orientation of the carbonate crystals in the living and not completely dried KR biofilm sample. Living (A) and a section of the KR biofilm sample stored in the refrigerator (9 °C) after 2 years (not completely dried sample) (B), where it is clearly visible that the vertically oriented mineral grains are composed of fibres and these span the layers of the biofilm (continuity is also visible); stereomicroscopic KRA part of the KR biofilm sample (C) and close view of the boxed part in C image indicates that the crystals are arranged by filamentous bacterial cells (D).

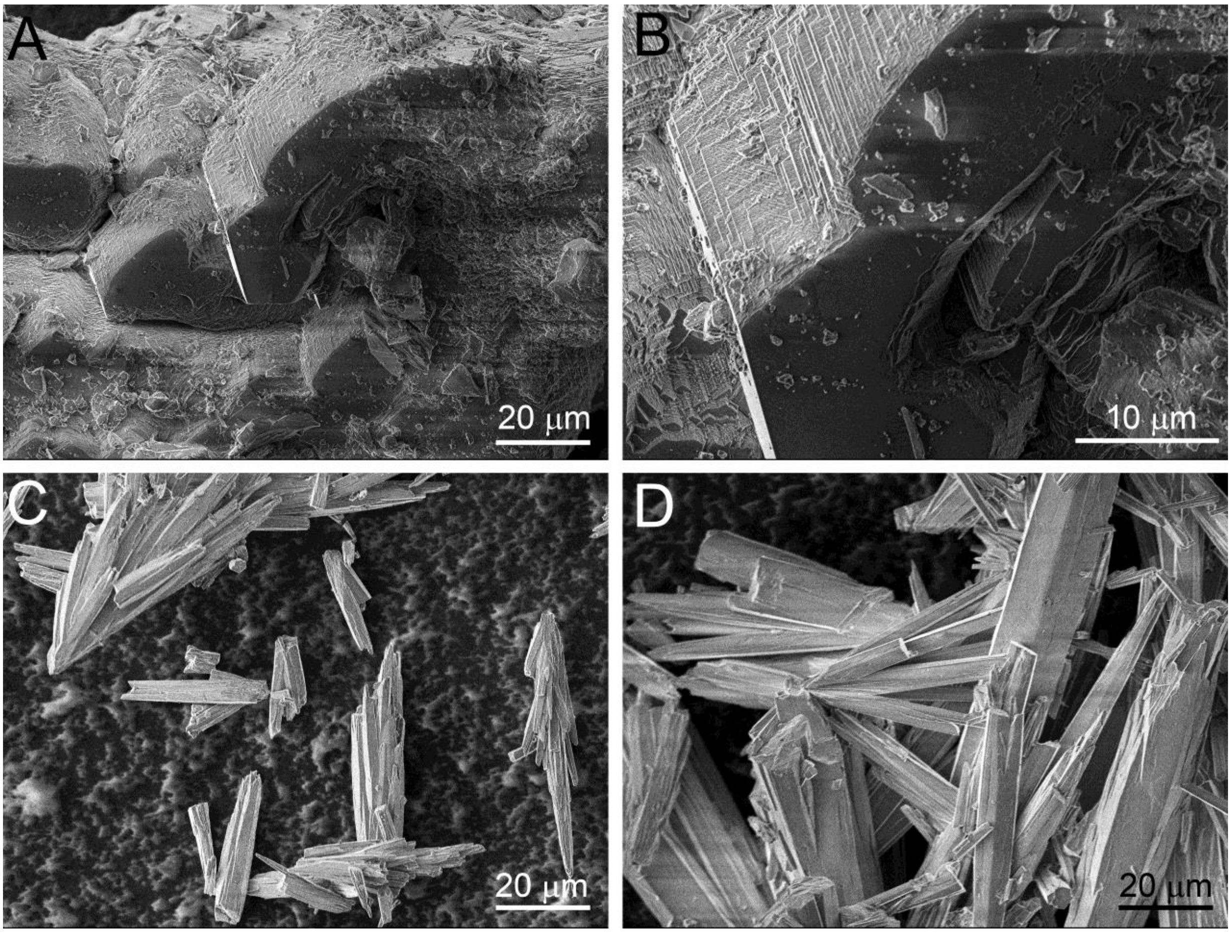

Supplementary Fig. S3. SEM images of euhedral calcite (A, B) and aragonite (C, D) crystals from sample points K2 (A, B), K3 (C) and K4 (D). Panel B was magnified from A.

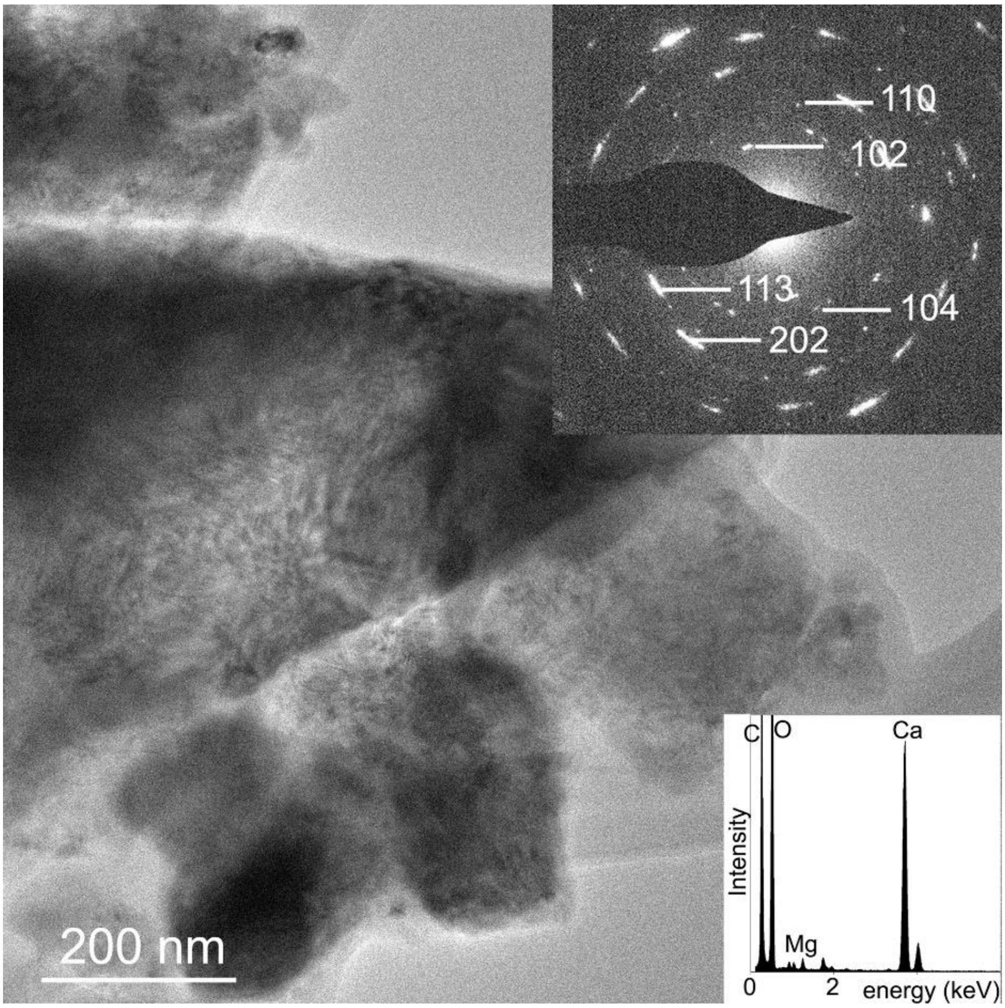

Supplementary Fig. S4. TEM data of nanocrystalline calcite from the KRD sample.

Supplementary Note 1. The composition of the microbial communities of the Köröm thermal well

Analysis of the next-generation sequencing data clarified that the water, carbonate precipitates and biofilms habitats of the Köröm thermal well were suitable environments for the colonization of different bacterial groups (Figs. 7, 8). In the well water the hydrogen-oxidizing genus *Hydrogenobacter* (Aquificota) was the most abundant. This genus contains extremophilic, obligate chemolithotrophic aerobic species, which utilizes H_2 as electron donor [1,2]. Other aerobic H_2 oxidizers from the genus *Hydrogenophilus* (phylum Pseudomonadota) [3] were also detected but with small abundance. Their abundance increased in the carbonate precipitate samples, where *Hydrogenobacter* decreased, probably due to the competition for the limited H_2. In the carbonate precipitates, unclassified Bacteria, the genera *Meiothermus* (Deinococcota) and *Tepidimonas* (Pseudomonadota) were dominant. The genus *Tepidimonas* contains chemolithoheterotrophic species, which require reduced sulfuric compounds for their metabolism.

The KRA biofilm layer contained a large proportion of mainly aerobic chemoorganotrophs, i.e., organisms capable to degrade biomacromolecules, belonging to the order Cytophagales.The genera *Hyphomonas*, *Amphiplicatus* and uncultured members of the family Sphingomonadaceae (Pseudomonadota) were also present in the KR samples. The genus *Hyphomonas* consists of aerobic, chemoorganotrophic organisms that are known to form benthic biofilms in marine ecosystems [4]. Members of the genus *Amphiplicatus* are obligate aerobic thermophilic bacteria. The family Sphingomonadaceae consists of strictly aerobic organisms with the exception of the genus *Zymomonas*, which has a phototrophic or chemoheterotrophic metabolism [5]. The aerobic organotrophic genus *Raineya* (Bacteroidota) was present in both layers of the green microbial biofilm. The class Oxyphotobacteria and other cyanobacterial groups in Köröm well promote aerobic metabolism, resulting in elevated oxygen concentrations during photosynthetically active periods within the colonised biofilm. Consequently, the characteristic anoxic segments of the biofilm were missing from both biofilm samples. This taxon diverged from its ancestor (2.5-2.6 bya) before the Great Oxygenation Event (2.4-2.1 bya), when it contributed greatly to the rise of the atmospheric oxygen levels [6]. The only obligate anaerobic, thermophilic genus *Pseudoclostridium* (Bacillota) [7] was present in both layers of the green microbial biofilm.

Previously, some representatives of *Hyphomonas* were isolated from oxygen rich deep sea hydrothermal effluents [4,8]. The first described species of *Amphiplicatus* was also discovered in a hot spring, located in northwestern China [9].

Some filamentous members of the genus *Meiothermus* capable of (red, orange, yellow) pigment production have been previously described from geothermal areas and marine hot springs [10,11]. The relative abundance of the phyla Bacteroidota, Cyanobacteria and Pseudomonadota was high in all the five layers of the red biofilm (KR). Members of the genus *Saprospira* (Bacteroidota), characteristic of the red biofilm layers (except for the KRA), are known to form long, colourful multicellular filaments, the colouration resulting from variable carotenoid pigments of pink, yellow, orange and red [12]. Due to the abundant pigment in Cytophagales cells, they often form yellow, orange, pink or red colonies [13], so their presence might explain the bright red colour of the KRA layer. The unusual mixed (orange/green) colour of the KRB biofilm layer could be associated with the high abundance of the strictly aerobic, thermophilic, orange colony-forming genus *Schleiferia thermophila* [14] together with the blue-green cyanobacterial filaments. The genus *Thermoflexibacter* (formerly genus *Flexibacter*) appeared only in the upper KRA and KRB red biofilm layers. The type strain of this genus has been described from a hydrothermal area in Iceland [15]. Some of the filaments detected by SEM in the Köröm red biofilm layers could be formed by this filamentous taxon, which is known for its unusually long filament formation [16]. The prominent green colour of the KRC and KRD samples can result from the high relative abundance of cyanobacterial taxa (unc. Oxyphotobacteria, *Glaeomargarita* and *Geitlerinema*).

In the green biofilm, the genus *Geitlerinema* was also one of the most abundant Cyanobacteria and is probably responsible for the deep green colouration and the oxygenation of KGA and KGB layers. The filamentous arrangement of the cells and extracellular mucus typically produced by this genus [17] contributed to the structural integrity of the green biofilm layers. These cyanobacteria have also been characterized as forming assemblages in dense associations that protect the microbial mat from the UV radiation in a natural ecosystem [18]. Similar densely interconnected filamentous microbial structures were typical structures in the uppermost layers of biofilms as revealed by electron microscopy analysis (Fig. 5C-E). The type species *Raineya orbicola* is known to develop colonies with orange pigmentation and its rod shaped cells can form long filaments during the exponential growth phase [19]. The presence of pigmented genera should also protect microbial mats from UV radiation, which can be detrimental for bacterial growth if exposed to high levels.

The genus *Raineya* and the class Bacteroidia were detected only in the K2 precipitate sample besides the biofilms. The overall community of K2 showed the highest degree of similarity to the red microbialite (KR). The similarity between the communities and the carbonate compositions of K2 and KR may be due to the fact that both were located outside the main water flow path, close to the air-water interface, in a sheltered area. The genus *Bacillus* (Bacillota) was detected in the K4 community and it has representative species known for their involvement in

carbonate mineral precipitation through their strong urease activity [20]. An aerobic *Bacillus* strain, isolated from an Italian shallow hydrothermal vent, was even capable of EPS production near 70 ^{o}C [21]. Some members of *Tepidimonas* are known to adsorb Ca^{2+} ions to their extracellular cell surfaces, thereby promoting $CaCO_3$ precipitation. Surface proteins of *Tepidimonas fonticaldi* AT-A2 have Ca^{2+} ions adsorption maximum under extremophilic conditions (150 ^{o}C) [22]. The most abundant, identified members of the phylum Cyanobacteria were the unc. Oxyphotobacteria with the genera *Geitlerinema* and *Gloeomargarita* in each red biofilm layer. Cyanobacteria are major contributors to $CaCO_3$ precipitation. They can promote carbonate deposition in several ways. Photosynthetic uptake of CO_2 in the form of HCO_3^- increases the local pH and the CO_3^{2-} saturation level in the proximity of the cells, thereby promoting induced calcium carbonate precipitation [23]. In addition, the structure and composition of the cell surface, S-layer and EPS can serve as nucleation sites for minerals, affecting carbonate precipitation [24]. The genus *Gloeomargarita* includes unicellular organisms even capable of precipitating intracellular amorphous calcium carbonate [25]. The thermophilic members of *Gloeomargarita* were also detected during metagenomic analysis of the bacterial communities from multiple hot springs in the Yellowstone National Park (YNP) [26]. It has been shown by cultivation methods that communities dominated by the filamentous members of the genus *Geitlerinema* produce stratified microbialites (stromatolites), where the carbonate precipitation is favoured by the extensive EPS secretion and the photosynthetic activity of the cyanobacteria.

Although many archaeal species can tolerate extremely high temperatures, amplicon sequencing shows that very few archaea are found in microbialite-like environments [27–29]. The archaeal community of the Köröm thermal well was dominated by the phylum Crenarchaeota, which included the ammonia-oxidizers (*C.* Nitrosotenuis, *C.* Nitrosocaldus, *C.* Nitrosotalea, *Nitrosarchaeum*) and their thermophilic representatives. The metabolic activity of these organisms contributed to the biogeochemical cycling of the nitrogen in the well water. The presence of the genus *Pyrobaculum* (Supplementary Table S5) may be explained by the ability of some species to reduce arsenate (*Pyrobaculum yellowstonensis* [30]; *Pyrobaculum arsenaticum* [31]), thus adapting to the high arsenic content of the Köröm thermal well.

Supplementary Note 2. The microbial community of the Köröm thermal well compared to recent hot spring biofilms

The structural and microbial community composition of microbial mats from the Köröm thermal well was distinct from active microbial mats formed in hot springs such as those in Yellowstone National Park (USA) [26,32,33], in Central Italy [34] and in the Baikal rift zone (Russia) [35]. The 15 hot springs studied in Yellowstone National Park had water temperature higher than 60 °C and a slightly alkaline pH [26]. Interestingly, the springs with the highest water temperatures (~70 °C) had green filamentous mats, whereas the lower temperature springs had orange-yellow microbial mats. The water temperature-dependent diversification could also be observed in the Köröm thermal well, where small patches of green mats were detected in the immediate vicinity of the well, whereas red microbial mats were found slightly further away from the well (Fig. 1B). Metagenomic sequencing results on the Illumina HiSeq 2500 platform showed that the dominant cyanobacterial taxon in Yellowstone National Park hot springs was the genus *Synechococcus*, which was not detected in the Köröm well samples. The genus *Gloeomargarita* was also present in some of the Yellowstone National Park hot springs as well, but with much lower abundance than in the Köröm well. Interestingly, the sampled microbial mats from the Yellowstone National Park did not contain any visible carbonate or other mineral precipitations [26].

In the Mammoth Hot Springs area of Yellowstone National Park, unique microbial mats have also been discovered along the hot spring outflow drainage systems. The Apron and Channel Facies (ACF) were covered by translucent to light beige *Sulfurihydrogenibium* (Aquificales) dominated microbial filaments and mats. The water temperature (60 -70°C) and pH (6.2-7.4) here were similar to the Köröm thermal water [32,33]. However, the surface morphology of the travertine and the carbonate precipitation processes observed in the microbial mat were significantly different from those observed in the area of the Köröm thermal well. At the ACF the rate of precipitation of travertine minerals was so high that the filamentous microbial mats were fully cemented to form a solid substrate just a few centimetres upstream from the non-encrusted filaments. The characteristic sinuous streamer fabric of the ACF travertine was explained by the encrustation of filamentous thermophilic bacteria by radial bundles of aragonite needles.

The hot spring systems in Central Italy had lower water temperature (≤ 55°C) than that of the Köröm well, the pH ranged from slightly acidic (6.37) to slightly alkaline (7.9). The initial sections of the vent channel contained microbial mats with mainly orange colouration, but the lower (~ neutral pH, and T >55 °C) sections were typically covered by green biofilms. This temperature dependent colouration was exactly opposite to that of the biofilms

from Köröm and the Yellowstone National Park. The community of these mats was fundamentally different from the similar coloured biofilms of Köröm thermal well based on NGS results focusing on the 16S rRNA gene's V3-V4 region (Illumina MiSeq platform). *Phormidium, Spirulina and Synechococcus* were the dominant cyanobacteria in the proximal orange mats, but *Synechococcus* was absent from the distal green mats. Sulphur-oxidizing and reducing bacteria as well as Chloroflexota were abundant in both segments. These taxonomic groups, which are closely connected to the high concentration of dissolved sulphur-compounds in the hot spring in Central Italy, were not detected in Köröm well. Carbonate (calcite spindles and aragonite spherulites) and iron oxide-hydroxide precipitations were observed in the biofilms by SEM and some of them also showed laminated ordering according to the bacterial filaments [34].

The Russian Garga hot spring located in the Baikal rift zone contains multiple cm-thick microbial mats on travertine depositions such as the Köröm green biofilm. The spring water temperature was 77 °C with a slightly alkaline pH (8.0-9.0). The thickest biofilms formed where the water temperature was below 70 °C. The dissolved sulfate concentration, however, was much higher (1000 mg/L) than in the Köröm thermal well. The phototrophic community of the Garga yellowish green biofilm mainly consisted of the phyla Cyanobacteria with the genera *Leptolyngbya, Synechococcus, Nostoc*, Chlorobiota and Chloroflexota with the genera *Chloroflexus, Anaerolinae* based on NGS sequencing of the 16S rRNA gene V3-V4 region (Illumina MiSeq platform). The abundance of anoxic phototrophs and obligate anaerobes increased in the deeper biofilm layers, indicating the presence of anoxic conditions in the proximity of the benthos. Heterothrophic colonizers from the phyla Pseudomonadota and Actinomycetota were also abundant in the biofilm. This microbial mat did not contain any visible carbonate or other mineral precipitations as well [35].

The large variation among the primary producers (e.g. oxygenic and anoxygenic photosynthesizers) could be related to the differences in the dissolved ion content of the separate water bodies. The water from the Köröm thermal well contained extremely high amount of dissolved arsenic (~ 400 µg/L), which should be a strong selective factor. In addition, the dissolved sulphur content of the Köröm well (33 mg/L) was much lower than would be required for bacterial sulphur biogeochemical cycling. The diverse archaeal community of the Garga hot spring resided mainly in the anoxic zone of the biofilm [35]. In contrast, the oxygen-producing photosynthesis of cyanobacteria detected in all layers of the Köröm well samples did not allow the formation of anoxic microenvironments. Only the traces of anoxygenic phototrophs (phylum Chloroflexota) were found in the biofilms from the Köröm thermal well in consistent with the low amounts of archaeal sequences (Supplementary Table S5).

All of these factors make the biofilms at the Köröm thermal well unique, and the results show that these structured biofilms can serve as starting templates for the building of shallow water microbialites.

References to Supplementary Note 1 and 2:

1. Pitulle, C. *et al.* Phylogenetic position of the genus *Hydrogenobacter*. *Int. J. Syst. Bacteriol.* **44**, 620–626 (1994).
2. Igarashi, Y. & Kodama, T. *Hydrogenobacter thermophilus*: its unusual physiological properties and phylogenic position in the microbial world. *FEMS Microbiol. Lett.* **87**, 403–406 (1990).
3. Manaia, C. M., Vaz-Moreira, I. & Nunes, O. C. *Hydrogenophilus*. in *Bergey's Manual of Systematics of Archaea and Bacteria* (eds. Trujillo, M. E. et al.) (Springer-Verlag, 2019).
4. Weiner, R. M., Devine, R., Powell, D. M., Dağaşan, L. & Moore, R. *Hyphomonas oceanitis* sp. nov., *Hyphomonas hirschiana* sp. nov., and *Hyphomonas jannaschiana* sp. nov. *Int. J. Syst. Becteriology* **35**, 237–243 (1985).
5. Yabuuchi, E. & Kosako, Y. Sphingomonadales ord. nov. in *Bergey's Manual of Systematics of Archaea and Bacteria* (eds. Trujillo, M. E. et al.) (Springer-Verlag, 2015).
6. Shih, P. M., Hemp, J., Ward, L. M., Matzke, N. J. & Fischer, W. W. Crown group Oxyphotobacteria postdate the rise of oxygen. *Geobiology* **15**, 19–29 (2017).
7. Zhang, X. *et al. Petroclostridium xylanilyticum* gen. Nov., sp. nov., a xylan-degrading bacterium isolated from an oilfield, and reclassification of clostridial cluster III members into four novel genera in a new Hungateiclostridiaceae fam. nov. *Int. J. Syst. Evol. Microbiol.* **68**, 3197–3211 (2018).
8. Jannasch, H. W. & Wirsen, C. O. Morphological survey of microbial mats near deep-sea thermal vents. *Appl. Environ. Microbiol.* **41**, 528–538 (1981).
9. Zhen-Li, Z. *et al. Amphiplicatus metriothermophilus* gen. nov., sp. nov., a thermotolerant alphaproteobacterium isolated from a hot spring. *Int. J. Syst. Evol. Microbiol.* **64**, 2805–2811 (2014).
10. Raulio, M. *et al.* Microbe repelling coated stainless steel analysed by field emission scanning electron microscopy and physicochemical methods. *J. Ind. Microbiol. Biotechnol.* **35**, 751–760 (2008).
11. Nobre, M. F. & da Costa, M. S. *Meiothermus*. in *Bergey's Manual of Systematics of Archaea and Bacteria* (eds. Trujillo, M. E. et al.) (Springer-Verlag, 2015).
12. Lewin, R. A. *Saprospira*. in *Bergey's Manual of Systematics of Archaea and Bacteria* (eds. Trujillo, M. E. et al.) (Springer-Verlag, 2015).

13. Nakagawa, Y. Cytophagales. in *Bergey's Manual of Systematics of Archaea and Bacteria* (eds. Trujillo, M. E. et al.) (Springer-Verlag, 2015).

14. Albuquerque, L., Rainey, F. A., Nobre, M. F. & da Costa, M. S. *Schleiferia thermophila* gen. nov., sp. nov., a slightly thermophilic bacterium of the phylum 'Bacteroidetes' and the proposal of Schleiferiaceae fam. nov. *Int. J. Syst. Evol. Microbiol.* **61**, 2450–2455 (2011).

15. Hahnke, R. L. *et al.* Genome-based taxonomic classification of Bacteroidetes. *Front. Microbiol.* **7**, (2016).

16. Lewin, R. A. A classification of Flexibacteria. *J. Gen. Microbiol.* **58**, 189–206 (1969).

17. Castenholz, R. W., Rippka, R. & Herdman, M. Form- *Geitlerinema* stat. nov. in *Bergey's Manual of Systematics of Archaea and Bacteria* (eds. Trujillo, M. E. et al.) (Springer-Verlag, 2015).

18. Popall, R. M., Bolhuis, H., Muyzer, G. & Sánchez-Román, M. Stromatolites as biosignatures of atmospheric oxygenation: carbonate biomineralization and UV-C resilience in a *Geitlerinema* sp. - dominated culture. *Front. Microbiol.* **11**, 948; 10.3389/fmicb.2020.00948 (2020).

19. Albuquerque, L. *et al. Raineya orbicola* gen. nov., sp. nov. a slightly thermophilic bacterium of the phylum bacteroidetes and the description of raineyaceae fam. nov. *Int. J. Syst. Evol. Microbiol.* **68**, 982–989 (2018).

20. Kim, H. J. *et al.* Calcium carbonate precipitation by *Bacillus* and *Sporosarcina* strains isolated from concrete and analysis of the bacterial community of concrete. *J. Microbiol. Biotechnol.* **26**, 540–548 (2016).

21. Nicolaus, B. *et al.* A thermophilic *Bacillus* isolated from an Eolian shallow hydrothermal vent, able to produce exopolysaccharides. *Syst. Appl. Microbiol.* **23**, 426–432 (2000).

22. Han, Y. L. *et al.* Calcium ion adsorption with extracellular proteins of thermophilic bacteria isolated from geothermal sites—A feasibility study. *Biochem. Eng. J.* **117**, 48–56 (2017).

23. Shiraishi, F. *et al.* Abiotic and biotic processes controlling travertine deposition: Insights from eight hot springs in Japan. *Sedimentology* **69**, 592–623 (2022).

24. Dupraz, C. *et al.* Processes of carbonate precipitation in modern microbial mats. *Earth-Science Rev.* **96**, 141–162 (2009).

25. Moreira, D. *et al.* Description of *Gloeomargarita lithophora* gen. nov., sp. nov., a thylakoid-bearing, basal-branching cyanobacterium with intracellular carbonates, and proposal for Gloeomargaritales ord. nov. *Int. J. Syst. Evol. Microbiol.* **67**, 653–658 (2017).

26. Kees, E. D., Murugapiran, S. K., Bennett, A. C. & Hamilton, T. L. Distribution and genomic variation of thermophilic cyanobacteria in diverse microbial mats at the upper temperature limits of photosynthesis. *mSystems* **7**, (5), ; 10.1128/msystems.00317-22 (2022).

27. Louyakis, A. S. *et al.* A study of the microbial spatial heterogeneity of bahamian thrombolites using molecular, biochemical, and stable isotope analyses. *Astrobiology* **17**, 413–430 (2017).

28. Roy, C. *et al.* Microbiome and ecology of a hot spring-microbialite system on the Trans-Himalayan Plateau. *Sci. Rep.* **10**, (1), 5917; 10.1038/s41598-020-62797- (2020).

29. Schuler, C. G., Havig, J. R. & Hamilton, T. L. Hot spring microbial community composition, morphology, and carbon fixation: Implications for interpreting the ancient rock record. *Front. Earth Sci.* **5**, 1–17 (2017).

30. Jay, Z. J. *et al. Pyrobaculum yellowstonensis* strain WP30 respires on elemental sulfur and/or arsenate in circumneutral sulfidic geothermal sediments of Yellowstone National Park. *Appl. Environ. Microbiol.* **81**, 5907–5916 (2015).

31. Huber, R., Sacher, M., Vollmann, A., Huber, H. & Rose, D. Respiration of arsenate and selenate by hyperthermophilic archaea. *Syst. Appl. Microbiol.* **23**, 305–314 (2000).

32. Fouke, B. W. Hot-spring Systems Geobiology: abiotic and biotic influences on travertine formation at Mammoth Hot Springs, Yellowstone National Park, USA. *Sedimentology* **58(1)**, 170–219 (2011).

33. Dong, Y. *et al.* Physiology, metabolism, and fossilization of hot-spring filamentous microbial mats. *Astrobiology* **19(12)**, 1442–1458 (2019).

34. Della Porta, G., Hoppert, M., Hallmann, C., Schneider, D. & Reitner, J. The influence of microbial mats on travertine precipitation in active hydrothermal systems (Central Italy). *Depos. Rec.* **8**, 165–209 (2022).

35. Rozanov, A. S., Bryanskaya, A. V., Ivanisenko, T. V., Malup, T. K. & Peltek, S. E. Biodiversity of the microbial mat of the Garga hot spring. *BMC Evol. Biol.* **17**, 254; 10.1186/s12862-017-1106-9 (2017).
